# Characterization of a Plant Nuclear Matrix Constituent Protein in Liverwort

**DOI:** 10.3389/fpls.2021.670306

**Published:** 2021-05-07

**Authors:** Nan Wang, Ezgi Süheyla Karaaslan, Natalie Faiss, Kenneth Wayne Berendzen, Chang Liu

**Affiliations:** ^1^Center for Plant Molecular Biology (ZMBP), University of Tübingen, Tübingen, Germany; ^2^Institute of Biology, University of Hohenheim, Stuttgart, Germany

**Keywords:** *Marchantia*, NMCP, nuclear periphery, nuclear lamina, nuclear envelope

## Abstract

The nuclear lamina (NL) is a complex network of nuclear lamins and lamina-associated nuclear membrane proteins, which scaffold the nucleus to maintain structural integrity. In animals, type V intermediate filaments are the main constituents of NL. Plant genomes do not encode any homologs of these intermediate filaments, yet plant nuclei contain lamina-like structures that are present in their nuclei. In *Arabidopsis thaliana*, CROWDED NUCLEI (CRWN), which are required for maintaining structural integrity of the nucleus and specific perinuclear chromatin anchoring, are strong candidates for plant lamin proteins. Recent studies revealed additional roles of *Arabidopsis* Nuclear Matrix Constituent Proteins (NMCPs) in modulating plants’ response to pathogen and abiotic stresses. However, detailed analyses of *Arabidopsis* NMCP activities are challenging due to the presence of multiple homologs and their functional redundancy. In this study, we investigated the sole *NMCP* gene in the liverwort *Marchantia polymorpha* (*MpNMCP*). We found that MpNMCP proteins preferentially were localized to the nuclear periphery. Using CRISPR/Cas9 techniques, we generated an *MpNMCP* loss-of-function mutant, which displayed reduced growth rate and curly thallus lobes. At an organelle level, *MpNMCP* mutants did not show any alteration in nuclear morphology. Transcriptome analyses indicated that *MpNMCP* was involved in regulating biotic and abiotic stress responses. Additionally, a highly repetitive genomic region on the male sex chromosome, which was preferentially tethered at the nuclear periphery in wild-type thalli, decondensed in the MpNMCP mutants and located in the nuclear interior. This perinuclear chromatin anchoring, however, was not directly controlled by MpNMCP. Altogether, our results unveiled that *NMCP* in plants have conserved functions in modulating stress responses.

## Introduction

The nuclear lamina (NL) is a fibrillar network that is composed of intermediate filaments and membrane-associated proteins, proving mechanical support to the nucleus (Dechat et al., 2008; Prokocimer et al., 2009). In addition, the NL also functions at the level of gene expression. Reports show that the NL serves as an attachment surface for chromosomes where it provides a repressive environment for gene silencing (Shevelyov and Nurminsky, 2012; van Steensel and Belmont, 2017). Accordingly, transcriptional activation of genes interacting with the NL correlates with local chromatin rewiring and NL detachment (Brueckner et al., 2020). For instance, in mammals and nematodes, X chromosome inactivation is mediated partially by tethering to the NL (Crane et al., 2015), which is dependent on the Lamin B receptor in mammals (Chen et al., 2016; van Steensel and Belmont, 2017). NL–chromatin interactions also contribute to genome organization. In eukaryotic nuclei, active chromatin and inactive chromatin occupy physically separated nuclear compartments (Zheng et al., 2018; Ulianov et al., 2019; Chang et al., 2020). Recent reports with various animal model systems revealed that deficiency in nuclear lamina proteins gave rise to increased chromatin interactions between active and inactive compartments (Zheng et al., 2018; Ulianov et al., 2019; Chang et al., 2020).

Animal nuclear lamins are type V intermediate filaments (Herrmann et al., 2009; Dittmer and Misteli, 2011). Plants do not have lamin-like analogs; however, there exists a lamina-like structure that helps to maintain nuclear morphology in the plant nucleus (McNulty and Saunders, 1992; Minguez and Moreno Diaz de la Espina, 1993; Masuda et al., 1997). Key components of this structure are a group of proteins known as nuclear matrix constituent proteins (NMCPs), which are plant-specific and highly conserved in the plant kingdom (Ciska et al., 2013). NMCPs are diverged into two phylogenetic groups, NMCP1 and NMCP2 (Ciska et al., 2019). In *Arabidopsis thaliana*, CROWDED NUCLEI (CRWN) proteins are the four NMCPs annotated as lamin candidates (Wang et al., 2013; Mikulski et al., 2019), among which CRWN1 to 3 and CRWN4 are classified as NMCP1 and NMCP2 type, respectively (Tamura et al., 2015). Functional analyses of CRWNs indicate their conserved function in regulating nuclear morphology as animal lamins (Zwerger et al., 2011; Tamura et al., 2015; Meier et al., 2016). At the cellular level, single *crwn1* and *crwn4* mutants show reduced nuclear size, and this phenotype is more pronounced in higher-order *crwn* mutants (Sakamoto and Takagi, 2013; Wang et al., 2013; Goto et al., 2014). Although individual single *crwn* growth similarly as wild-type (WT) plants, a low but non-trivial level of *CRWN* activities is required to complete the plant life cycle, as mutants lacking all the four *CRWN* genes are inviable (Wang et al., 2013).

*Arabidopsis CRWN* genes, especially *CRWN1*, are so far the most intensively investigated plant *NMCP*. In addition to regulating nuclear size and morphology, *CRWN1* has also been found to be involved in protecting DNA against oxidative damage (Wang et al., 2019), pathogen response (Guo et al., 2017; Choi et al., 2019; Jarad et al., 2019), promoting copper tolerance (Sakamoto et al., 2020), ABA signaling pathway (Zhao et al., 2016), and chromatin activities. CRWN1 and CRWN4 proteins are localized at the nuclear periphery, where they are both involved in regulating specific perinuclear chromatin anchoring (Sakamoto and Takagi, 2013; Poulet et al., 2017b; Hu et al., 2019). CRWN1 was shown recently to tether repressed chromatin domains to the NL by binding to inaccessible chromatin (Hu et al., 2019). Furthermore, CRWN1 and CRWN4 play a role in regulating heterochromatin integrity and condensation (Wang et al., 2013; Poulet et al., 2017a). Additionally, CRWN is also able to mediate active gene expression. A recent report by Sakamoto and colleagues shows that CRWN1 participates in positioning copper tolerance genes at the NL and mediating their transcriptional upregulation under excess copper conditions (Sakamoto et al., 2020). With the use of proteomic approaches, CRWN1 has been shown to interact with transcriptional regulators, such as Polycomb Repressive Complex 2-associated factor PWO1 (Mikulski et al., 2019) and NAC transcription factor NTL9 (Guo et al., 2017). A CRWN homolog in rice, OsNMCP1, has been found to interact with a subunit belonging to SWITCH/SUCROSE NON-FERMENTING (SWI/SNF) chromatin remodeling complex to modulate drought stress response (Yang et al., 2020).

Studies of multiple *Arabidopsis CRWN* genes also unveiled their functional redundancy in suppressing autoimmunity. RNA sequencing (RNA-seq) experiments of several *crwn* single and double mutants indicated that the gene expression profile of these mutants was concomitant with the activation of pathways related to plant–pathogen interactions, for example, in the activation of *PR1* (PATHOGENESIS-RELATED1) and *PR2* and *PR5* expression (Guo et al., 2017; Choi et al., 2019). With ectopically activated biotic stress response pathways, *crwn1 crwn2* and *crwn1 crwn4* double mutants showed greater resistance to infection with virulent bacterial pathogens (Guo et al., 2017; Choi et al., 2019).

The liverwort *Marchantia polymorpha* is a dioecious non-vascular plant with a mostly haploid life cycle and a highly streamlined genome: small size (ca. 280 Mbp) with low genetic redundancy (Tanurdzic and Banks, 2004). Early phylogenetic analysis indicates that the *Marchantia* genome possesses only one NMCP homolog (hereafter referred to as “*MpNMCP*”) (Ciska et al., 2019). It is not known if this sole *NMCP* gene in *Marchantia* shares conserved functions as those discovered in higher plants. In this study, we report that *MpNMCP* is a lamina-like nuclear periphery protein. We found that while the growth of *Mpnmcp* mutants was suppressed, loss of *NMCP* did not block vegetative growth of *Marchantia*, and *Mpnmcp* mutant nuclei did not show altered nuclear size or shape. We also found that a heterochromatic region from the *Marchantia* male sex chromosome displayed preferential perinuclear localization, but it detached from the NL in *Mpnmcp* nuclei and decondensed. However, MpNMCP proteins did not show direct interactions with chromatin at the nuclear periphery. Nevertheless, transcriptome experiments indicated that *MpNMCP* was involved in suppressing ectopic stress responses. Overall, the *Marchantia NMCP* has remarkable differences in functional conservation compared with *NMCPs* in higher vascular plants, except the sphere of plant stress responses, where it retains a similar function.

## Results

### *MpNMCP* Promotes Basic Growth of *Marchantia*

The *Marchantia* genome assembly has been recently upgraded from a scaffold (v3.1) to chromosomal scale version (v5.1) containing slightly more annotated genes (Montgomery et al., 2020). Thus, we performed a homology search to check if there were additional *Marchantia* NMCP homologs missed in the earlier phylogenetic analyses using the older genome assembly. The protein sequences of *Arabidopsis* CRWN1 and another two NMCPs from *Physcomitrella patens* were used to search for *Marchantia* NMCP homologs with Basic Local Alignment Search Tool for protein sequences (BLASTp). For each of the inquiry sequences, only one hit was found, confirming that the *Marchantia* genome encodes only one putative NMCP (Supplementary Figure 1; Ciska et al., 2019).

**FIGURE 1:**
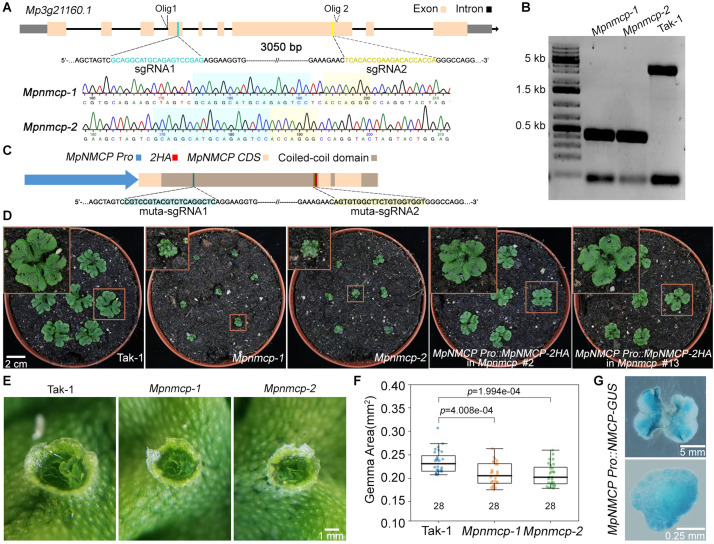
*Mpnmcp* mutants show dwarf phenotype. **(A)** Schematic depiction of the *MpNMCP* locus and single-guide RNAs (sgRNAs) aiming for site-directed mutagenesis (top). Representative sequencing tracks of using Olig1 (Supplementary Table 2) showing the deletion of a ∼3-kb region flanked by the two sgRNAs (middle and bottom). **(B)** Genotyping PCR results of individual T3 *Mpnmcp* mutants. Genotyping was carried out by using genotype specific primers: Olig1 (forward primer) and Olig2 (reverse primer) (primer sequences are given in Supplementary Table 2). **(C)** Schematic depiction of the *MpNMCP CDS* with *MpNMCP* promoter (2-kb upstream of the gene model). HA, hemagglutinin tag. muta-sgRNA1 and muta-sgRNA2 indicate the two regions to which silent mutations are introduced to avoid recognition by the sgRNAs used for site-directed mutagenesis of the endogenous *MpNMCP* locus. **(D)** Phenotype of 3-week-old wild-type (WT) Tak-1, *Mpnmcp* mutants, and two independent *MpNMCP Pro:MpNMCP-2HA/Mpnmcp* transgenic lines. The *MpNMCP Pro:MpNMCP-2HA* in the *Mpnmcp* mutant displayed normal development. **(E)** Close-up of one gemma cup from 20-day-old WT Tak-1 or *Mpnmcp* mutants, which have mature gemmae inside. **(F)** Boxplot shows the size of gemmae collected from gemma cups of *Mpnmcp* mutants and Tak-1 plants. *n* = 28. p values were determined by Mann–Whitney *U* test. **(G)** Glucuronidase (GUS) staining of *MpNMCP Pro:GUS* plants. Pictures show the representative distribution pattern of GUS staining in 2-week-old thallus (top panel, scale bar = 5 mm) and gemmae (bottom panel, scale bar = 0.25 mm).

Next, to clarify the function of *MpNMCP*, we generated *Mpnmcp* mutants using the CRISPR/Cas9 system, by which two guide RNAs were designed to delete most of MpNMCP’s coding region (Figure 1A). All T1 mutant lines carrying a deletion allele displayed severely delayed thallus growth; among them, we chose two lines in the Takaragaike-1 (Tak-1) background, namely, *Mpnmcp-1* and *Mpnmcp-2*, for further phenotype characterization. Sequencing of these mutants revealed that both of them had a ∼3-kb deletion between the two single-guide RNAs (sgRNAs) (Figures 1A,B). Under a standard greenhouse condition, *Mpnmcp* mutant thalli showed dwarf phenotypes and curly branches as compared with the WT Tak-1 plants (Figure 1D). Asexual reproduction of *Mpnmcp* mutants was largely unaffected: they could produce gemma cups similarly to WT Tak-1 thalli (Figure 1E). Smaller gemmae, however, were found within these gemmae cups (Figure 1F).

To confirm that the mentioned *Mpnmcp* phenotypes were causal to the lack of functional *MpNMCP*, we generated complementation lines by double transformation, in which the WT *MpNMCP* coding sequence fused with a tandem hemagglutinin (HA) tag driven by the native *MpNMCP* promoter and introduced it into null *Mpnmcp* mutant background (Figure 1C). The growth defects exhibited by the Mpnmcp mutants were largely rescued in the complementation lines (Figure 1D and Supplementary Figure 10). Altogether, phenotypic characterization of *Mpnmcp* mutants revealed that the absence of *MpNMCP* leads to general growth repression.

To characterize *MpNMCP* spatial–temporal expression patterns in greater detail, we generated reporter lines in which the same *MpNMCP* promoter region (Figure 1C) was placed in front of the *beta*-glucuronidase (GUS) reporter coding sequence. We analyzed GUS staining patterns in more than 30 independent transgenic *MpNMCP Pro:GUS* lines, all of which showed GUS activity throughout thalli except for older parts at thallus margins (Figure 1G). In gemmae, the GUS staining was visible across the entire sample (Figure 1G).

### *MpNMCP* Suppresses Ectopic Onset of Stress Response

**FIGURE 2:**
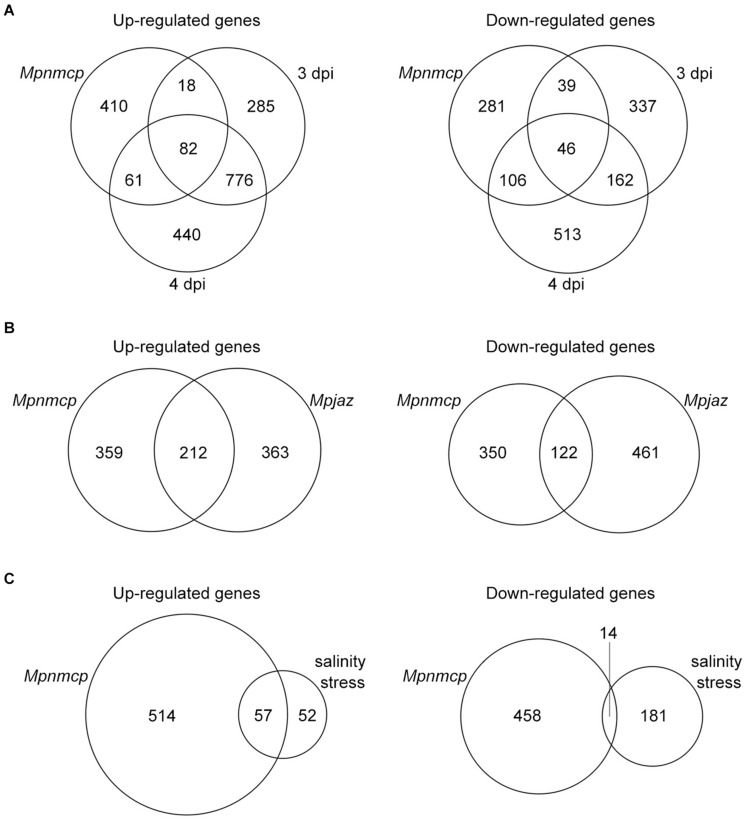
Characteristics of differentially expressed genes (DEGs). **(A)** Venn diagram showing the overlap of DEGs among *Mpnmcp*, 3 dpi (days post inoculation with *Phytophthora palmivora*) and 4 dpi. **(B)** Venn diagram showing the overlap of DEGs between *Mpnmcp* and *Mpjaz*. **(C)** Venn diagram showing the overlap of DEGs between *Mpnmcp* and *Marchantia* under salinity stress.

To gain a better understanding of how *MpNMCP* regulates thallus growth, we performed RNA-seq to compare the gene expression profile of 2-week-old *Mpnmcp* mutant and WT thallus tissues. In total, 571 and 472 genes were annotated as up- and downregulated genes shared between two independent mutant lines, respectively (Supplementary Figure 2 and Supplementary Table 1). As the function of *NMCP* genes in both *Arabidopsis* and rice has been linked to stress response (Guo et al., 2017; Choi et al., 2019; Jarad et al., 2019; Yang et al., 2020), we postulated that the *Mpnmcp* mutant transcriptome would show correlation to those stress conditions. To this end, we compared differentially expressed genes (DEGs) in *Mpnmcp* mutants and several published stress-related *Marchantia* transcriptomes. To capture pathogen response, we used a transcriptome dataset of *Marchantia* infected by hemi-biotrophic oomycete *Phytophthora palmivora* (Carella et al., 2019). Among the 571 upregulated genes in *Mpnmcp*, 100 (*p* = 1.02e–24, Fisher’s exact test) and 143 (*p* = 6.03e–46, Fisher’s exact test) were upregulated in 3-dpi (days post inoculation) and 4-dpi samples, respectively (Figure 2A). A similar extent of overlap was found for downregulated genes, where 85 (*p* = 3.20e–44, Fisher’s exact test) and 152 (*p* = 1.19e–96, Fisher’s exact test) downregulated genes in *Mpnmcp* showed suppressed expression in 3- and 4-dpi samples, respectively (Figure 2A). The jasmonate (JA) pathway has recently been shown as a suppressor of defense in *Marchantia* (Monte et al., 2019; Matsui et al., 2020). Interestingly, for the *Mpjaz* mutant that exhibits constitutive activation of the JA pathway, its transcriptome also resembled that of the *Mpnmcp* mutant (Figure 2B). However, only one of the three JA maker genes *MpAOS2* (*Mp5g16260*), *MpAOC* (*Mp7g06220*), and *MpCHL* (*Mp8g10460*), which were highly upregulated in *Mpjaz* (Monte et al., 2019), showed noticeable upregulation in *Mpnmcp*, suggesting a partially activated JA pathway in our mutants (Supplementary Figure 3). We also compared *Mpnmcp* transcriptome with a salt-stress transcriptome dataset (Tanaka et al., 2018). Due to a lower sequencing coverage of this salt-stress transcriptome dataset and our filter settings of DEG calling, there were only 109 genes annotated as salt-stress responsive. Nevertheless, more than half of them (*p* = 2.787e–62, Fisher’s exact test) were upregulated in the *Mpnmcp* mutant (Figure 2C). Altogether, the noticeable overlap of DEGs between the *Mpnmcp* mutant and different stress-related transcriptome suggests that the role of plant *NMCP* genes in regulating stress response is conserved in *Marchantia*.

### Mpnmcp Is Localized at the Nuclear Periphery but Does Not Control Nuclear Morphology

**FIGURE 3:**
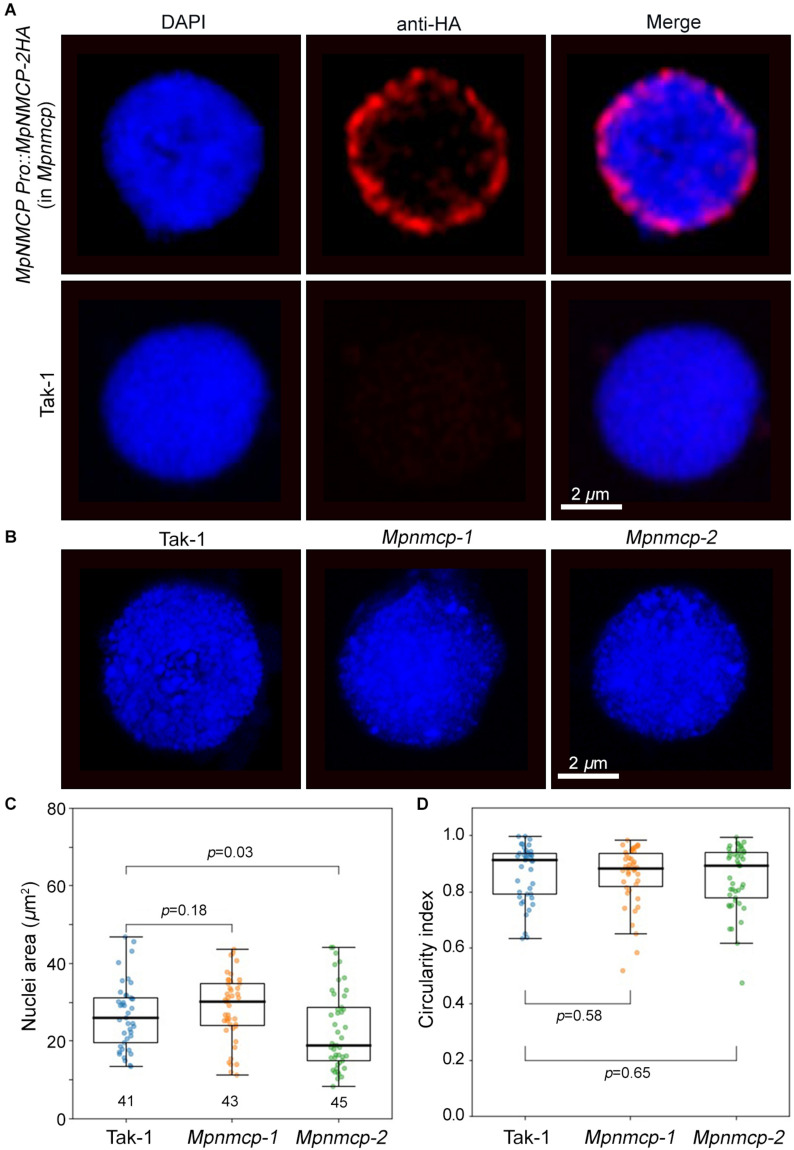
*Mpnmcp* mutants do not show observable defects in nuclear morphology. **(A)** Immunohistostaining shows perinuclear localization of MpNMCP:2HA proteins (red, top panel). The image shown in the bottom panels serves as a negative control. 4′,6-Diamidino-2-phenylindole (DAPI) staining (blue) was used for visualizing nuclei. Scale bar shows 2 μm. **(B)** Representative Takaragaike-1 (Tak-1) (left) and *Mpnmcp* (middle and right-hand panels) nuclei stained with DAPI. **(C,D)** Comparison of nuclear morphology. For each genotype, measurements were taken from the maximum z-projected images of around 45 nuclei collected from gemmae to compute their nuclear size (C) and circularity index **(D)**. p values were determined using Mann–Whitney *U* test.

We next examined nuclear localization patterns of MpNMCP proteins by immunohistostaining, in which functional MpNMCP:2HA fusion proteins were visualized with fluorophore-conjugated anti-HA antibodies. As shown by representative images in Figure 3A, MpNMCP is preferentially localized at the nuclear periphery *in vivo*, which is similar to many NMCP proteins from various plants species (Ciska et al., 2013; Sakamoto and Takagi, 2013; Yang et al., 2020; McKenna et al., 2021). Besides, we found that MpNMCP proteins were not evenly distributed at the nuclear periphery but exhibited a punctate-like pattern, which was similar to that from onion (Ciska et al., 2013).

At the nuclear periphery, *Arabidopsis* NMCPs CRWN1 and CRWN4 are required to maintain nuclear size and shape; mutant plants losing either *CRWN1* or *CRWN4* show smaller and round nuclei (Wang et al., 2013). Additionally, overexpression of *Arabidopsis CRWN1* can partially deform the nuclear envelope by forming invaginations (Goto et al., 2014). Similar effects with the maize NMCP homolog, *NCH1*, have also been reported recently (McKenna et al., 2021). The conserved localization of *Marchantia* NMCP at the NL prompted us to ask if knocking out *MpCRWN* would result in similar defects in nuclear morphology and whether these defects would underlie the suppressed growth phenotype seen in the *Mpnmcp* mutants. After quantifying nuclear size and shape, we were not able to detect any differences between *Mpnmcp-1* and WT Tak-1 (Figures 3C,D). However, we found that *Mpnmcp-2* mutant had smaller nuclei (*p* = 0.03, Mann–Whitney *U* test) (Figure 3C). As the two mutant lines did not display consistent changes in nuclear size, we conclude that losing *MpNMCP* does not cause an effect on nuclear size.

**FIGURE 4:**
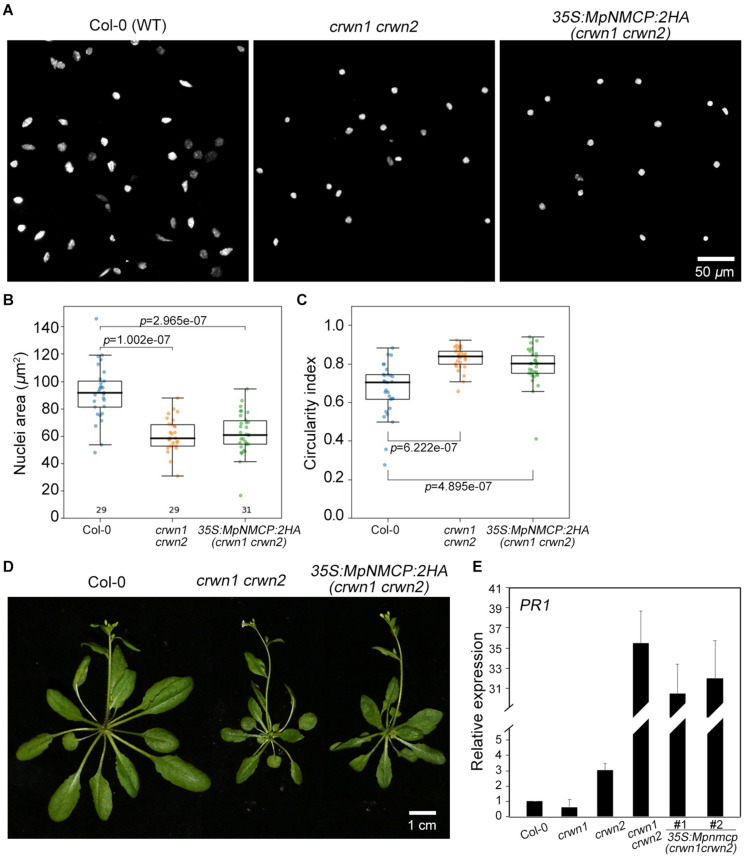
*MpNMCP* could not complement *Arabidopsis crwn* mutants. **(A)** Comparison of 8C nuclei morphology. The nuclei were isolated from the first true leaf of 2-week-old seedlings. 4′,6-Diamidino-2-phenylindole (DAPI) staining (blue) was used for visualization of nuclei. Scale bar, 50 μm. **(B,C)** Comparison of nuclear morphology. Quantifications of nuclear size **(B)** and nuclear circularity index (C) in Col-0, *crwn1 crwn2*, and *crwn1 crwn2 35S:MpNMCP-2HA*. **(D)** Phenotypes of transgenic *Arabidopsis* plants over-expressing *MpNMCP:2HA*. Scale bar, 1 cm. **(E)** The relative expression level of *PR1* in different mutants detected by real-time PCR. Values were normalized to the expression of *ubiquitin 5* (*UBQ5*). Error bars display ± SD. *PR1* expression in individual biological replicates is set as 1.

To further examine if MpNMCP proteins possess abilities in shaping nuclei in higher plants, we exogenously over-expressed *MpNMCP* in the *Arabidopsis crwn1 crwn2* double mutant that shows nuclear morphology changes. Loss-of-function of both *CRWN1* and *CRWN2* had synergistic effects that give rise to much smaller nuclei than *crwn1* or *crwn2* single mutants (Wang et al., 2013). We examined over 20 independent lines of *crwn1 crwn2 35S:MpNMCP:2HA* plants that had confirmed *MpNMCP:2HA* transgene expression (Supplementary Figure 11). We found that all of them developed nuclei with similar morphology compared with *crwn1 crwn2*, indicating that *MpNMCP* could not regulate nuclear shape or size in *Arabidopsis* (Figures 4A–C). In addition, *crwn1 crwn2 35S:MpNMCP:2HA* seedlings phenocopied *crwn1 crwn2* double mutants that showed growth retardation and dwarfism (Figure 4D). This growth phenotype has been related to ectopic activation of biotic stress response (Guo et al., 2017; Choi et al., 2019) part of which involves a hyperactive salicylic acid pathway.

As our transcriptome analyses revealed a connection between *MpNMCP* and biotic stress-signaling pathway in *Marchantia* (Figure 2), we checked if *MpNMCP* could partially suppress salicylic acid pathway as its *Arabidopsis* homologs *via* analyzing the *PR1* gene expression. Loss-of-function of *CRWN1* and *CRWN2* produces a synergistic effect in activating *PR1*. In 10-day-old *crwn1 crwn2* double-mutant seedlings, *PR1* was expressed approximately 35 times as high as in WT plants (Figure 4E). Compared with *crwn1 crwn2*, in *crwn1 crwn2 35S:NMCP:2HA*, only a subtle decrease in *PR1* expression was observed (Figure 4E). Together, these data indicate that *MpNMCP* could not functionally complement *Arabidopsis NMCPs*.

### V Chromosome Repeats Lost Perinuclear Localization and Decondense in *Mpnmcp*

**FIGURE 5:**
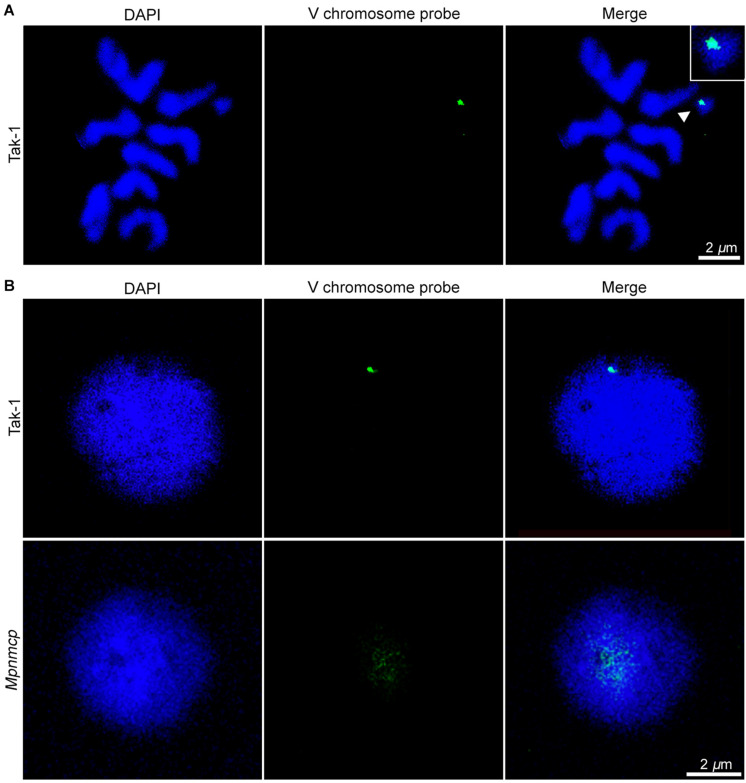
Fluorescence *in situ* hybridization (FISH) analysis of repeats from the V chromosome. **(A)** Chromosome spread confirms the specificity of FISH probes to the V chromosome (pointed by an arrowhead). The scale bar represents 2 μm. **(B)** Distribution of the probed genomic region from V chromosome in Tak-1 (top row) and *Mpnmcp mutant* (bottom row) nuclei. The nuclei were isolated from vegetative thalli fixed with 0.1% formaldehyde. The scale bar represents 2 μm. Nuclei are counterstained with 4′,6-diamidino-2-phenylindole (DAPI) (blue).

In addition to regulating nuclear morphology, *Arabidopsis* NMCPs also participates in perinuclear chromatin anchoring and heterochromatin organization (Wang et al., 2013; Poulet et al., 2017a; Hu et al., 2019). Our recent study on *Arabidopsis* CRWN1 revealed that it tethers repressed chromatin domains to the nuclear periphery *via* direct protein–chromatin interactions (Hu et al., 2019). We hypothesized that if MpNMCP proteins had such a function, *Marchantia* chromatin regions with preferential localization at the nuclear periphery would show detachment from the NL in *Mpnmcp* mutants. In most eukaryotic cells, heterochromatin regions are enriched at the nuclear periphery (Buchwalter et al., 2019). Across the *Marchantia* genome, the V chromosome stands out as a good candidate with potential perinuclear localization patterns, because it carries a much higher level of heterochromatic marks (e.g., H3K9me1 and H3K27me1) and repeats than autosomes (Montgomery et al., 2020). Thus, we used a probe derived from previously identified V chromosome-specific repeats, which had multiple copies located in the 6.4- to 7.5-Mb interval, to examine the nuclear location of the corresponding genomic regions (Okada et al., 2001). The specificity of the probe could be confirmed, as hybridization only occurred in the V chromosome (Figure 5A). With this probe, our fluorescence *in situ* hybridization (FISH) experiment on Tak-1 thallus nuclei showed preferential localization of the hybridization signal at the nuclear periphery (Figure 5B and Supplementary Figures 4A,B). At times, more than one signal spot was observed, probably reflecting the presence of multiple copies of genomic regions bearing probe sequences (Supplementary Figure 4B).

Surprisingly, we could not detect any hybridization signal in *Mpnmcp* mutant nuclei with our default FISH protocol (Supplementary Figure 4, third row). We ruled out the possibility that the target genome regions were lost in the *Mpnmcp* mutant, as the probe could produce positive signals when subjected to chromosome spread FISH (data not shown). Instead, the hybridization of probe to *Mpnmcp* nuclei appeared to be sensitive to sample preparation, in that a much lower concentration of formaldehyde had been used for nuclear fixation (Figure 5B). With a revised sample fixation procedure, we found that the target genomic regions in *Mpnmcp* adopted a decondensed configuration and were not positioned at the nuclear periphery, suggesting that *Marchantia NMCP* functions in perinuclear chromatin tethering (Figure 5B).

Next, we asked if the V chromosome as a whole had preferential localization at the nuclear periphery. As tiling bacterial artificial chromosome (BAC) clones covering the V chromosome were not available, we approximated V chromosome FISH by mixing labeled Tak-1 genomic DNA with excessive unlabeled Tak-2 genomic DNA so that the latter could suppress autosome DNA hybridizing with the probes. In the absence Tak-2 DNA, FISH signals were found throughout Tak-1 nuclei (Supplementary Figure 5A). By contrast, the addition of Tak-2 DNA resulted in specific signals occupying a subdomain in the Tak-1 nucleus, which presumably corresponded to the V chromosome territory (Supplementary Figure 5B). Overall, most of the V chromosome FISH signals did not show contacts with the nuclear periphery (Supplementary Figure 5B). In summary, perinuclear chromatin anchoring was limited to parts of the V chromosome.

### *Mpnmcp* Genome Does Not Show Drastic Conformational Changes

**FIGURE 6:**
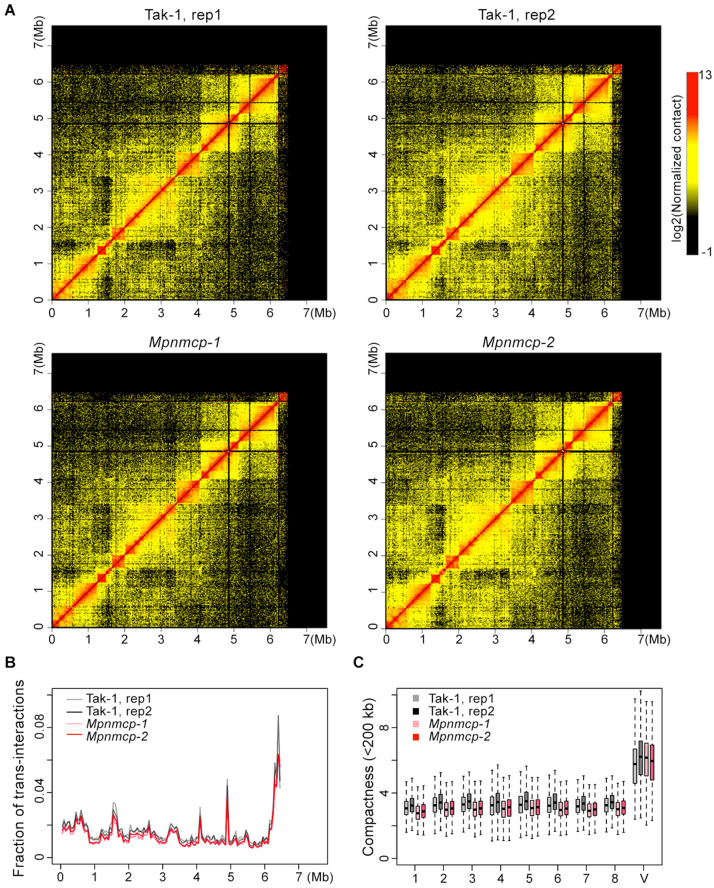
Comparison of the V chromosome organization patterns in wild-type (WT) Tak-1 and *Mpnmcp*. **(A)** Hi-C maps of the V chromosome normalized at 10-kb resolution. **(B)** Inter-chromosomal interaction patterns across the V chromosome. **(C)** Comparison of chromatin compactness of individual chromosomes derived from Hi-C maps.

Given conformational changes (decondensation) in the abovementioned repeat region at the V chromosome in *Mpnmcp* mutants, we performed Hi-C experiment to compare their genome-wide chromatin organization patterns with those of WT Tak-1 (Supplementary Figure 6). Because the repeat region (6.5–7.5 Mb) at the V chromosome had poor read mapping quality, it was masked during Hi-C map normalization. Therefore, we could not examine how the V chromosome chromatin packing pattern was or how it interacted with other genomic regions. The rest part of the V chromosome, based on manual inspection, did not show drastic conformational changes, including topologically associated domains (TADs) that we reported in an earlier study (Figure 6; Montgomery et al., 2020). We further performed quantitative analyses to assess possible chromatin packing changes. We reasoned that if the V chromosome in *Mpnmcp* became decondensed, it would gain more contacts with autosomes and show weaker long-range intra-chromosomal interactions. Compared with WT Tak-1, we found that genomic regions along the V chromosome in *Mpnmcp* did not exhibit an altered level of *trans*-interactions (Figure 6B). Additionally, we found that the V chromosome did not show decreased long-range intra-chromosomal interactions, which were approximated from compactness values (sum of *cis*-contacts up to 200 kb) from Hi-C map normalized at 10-kb resolution (Figure 6C). These results did not support our hypothesis that other parts at the V chromosome adapted a decondensed state in *Mpnmcp* mutants.

However, individual autosomes in *Mpnmcp* showed a mild drop in their compactness values, suggesting that they became slightly decondensed in the mutants (Figure 6C). We further calculated interaction decay exponents of the autosomes (up to 2.5 Mb), which was a measure of how fast chromatin *cis*-contacts drop with increasing genomic distance. Consistent with their compactness values, autosomes in *Mpnmcp* showed a slightly faster decay of chromatin contact strength as genomic distance increased (Supplementary Figure 7A). Nevertheless, such nuanced chromatin decondensation was not accompanied with elevated inter-chromosomal interactions at autosomes (Supplementary Figure 7B). Altogether, we conclude that in spite of having chromatin decondensation at the end of the V chromosome (approximately 6.5 to 7.5 Mb), lost-of-function of *MpNMCP* did not result in drastic alteration in genomic organization.

## Discussion

### MpNMCP Is Not Involved in Regulating Nuclear Morphology

In this study, we functionally characterized the sole NMCP homolog encoded by the *Marchantia* genome and compared it with AtCRWN1. While the MpNMCP proteins have similar domain architecture and identical nuclear localization patterns to AtCRWN1 (N-terminal conserved coiled-coil domains), it functions differently in liverwort.

Early functional characterization of AtCRWN1 unveiled its role in regulating nuclear morphology (Dittmer et al., 2007). Functional studies of other NMCPs have been conducted on multiple plants, including rice (Yang et al., 2020), maize (McKenna et al., 2021), and onion (Ciska et al., 2013); yet it is unknown whether the corresponding *NMCP* genes in these plants play a structural role in the nucleus. In this study, we showed that although MpNMCP proteins were located at the nuclear periphery (Figure 3A), the *Mpnmcp* mutant does not show changes in nuclear size or shape (Figures 3B–D). While measuring nuclear sizes, we found noticeable variation of data values (Figure 3C), and in our opinion, this was due to sampling of different cell types. At the moment, we do not have *Marchantia* cell-lineage maker lines that allow us to analyze nuclear morphology of a specific cell type. However, according to studies in *Arabidopsis*, the effect of losing CRWNs (i.e., smaller nuclei) can be found consistently in multiple cell types (Poulet et al., 2017a). Therefore, we expected a shift in nuclear size, regardless of cell type in *Mpnmcp* mutants if the *Marchantia* NMCP homolog is required for nuclear size regulation. As a statistically significant difference was only observed in one of the *Mpnmcp* mutant line (Figure 3C), we conclude that losing *MpNMCP* does not influence the nuclear size.

Furthermore, *MpNMCP* could not rescue the *Arabidopsis crwn1 crwn2* mutant phenotype (Figure 4A). It should be noted that the absence of functional complementation of *crwn1 crwn2* by *MpNMCP* might be attributed to other technical issues; for example, *Arabidopsis* plant cannot generate post-transcriptional modifications or homologous proteins that are important for MpNMCP to be fully functional. Nevertheless, together with nuclear morphology analysis on *Mpnmcp* mutants, these results suggest that the nuclear morphology in *Marchantia* is not controlled by *MpNMCP* but by other structural proteins. In higher plants, two other types of putative nuclear lamina-like genes that regulate nuclear size are *KAKU4* (Goto et al., 2014) and Nuclear Envelope-Associated Proteins (NEAPs) (Pawar et al., 2016). Our sequence analysis revealed that the *Marchantia* genome encodes no *KAKU4* homologs but one putative *NEAP* (*Mp4g20150*). It is therefore tempting to speculate a potential structural role of MpNEAP concerning nuclear morphology regulation.

### Conserved Function of MpNMCP in Stress Signaling

Both *Arabidopsis* and rice NMCP proteins have been found to be involved in stress signaling. AtCRWNs have also been shown to regulate transcriptional levels of stress and defense response genes (Guo et al., 2017; Choi et al., 2019; Choi and Richards, 2020). *crwn1 crwn2* and *crwn1 crwn4* mutants showed greater resistance to infection with virulent bacterial pathogens and increased *Pathogenesis-Related1* (*PR1*), *PR2*, and *PR5* transcript levels (Guo et al., 2017; Choi et al., 2019). *PR1* expression is inhibited by the NAC transcription factor-like 9 (NTL9). NTL9 interacts with AtCRWN1 and Suppressor of NPR1 inducible 1 (SNI1), possibly forming a protein complex to suppress *PR1* expression and inhibit the activity of NPR1, a master regulator of pathogen resistance in *Arabidopsis* (Kim et al., 2012; Guo et al., 2017). OsNMCP1 has also been associated with drought tolerance. Overexpression of *OsNMCP1* improves rice drought resistance and results in deeper and thicker root systems, which are of pivotal importance in alleviating water stress (Yang et al., 2020).

Our study reveals a connection between *MpNMCP* and stress response in *Marchantia*. First, by comparing DEGs in *Mpnmcp* mutants and several published stress-related *Marchantia* transcriptome datasets, we found a noticeable overlap between *Mpnmcp* DEGs and stress-related DEGs (Figure 2A). Second, we found transcriptome resemblance between *Mpnmcp* and *Mpjaz* mutants (Figure 2B). The expression of *MpCHL*, which is the precursor of JA biosynthesis (12-oxo-phytodienoic acid, OPDA)-marker gene, was strongly promoted (ca. 50-fold) in *Mpnmcp* (Supplementary Figure 3). Such gene upregulation, however, is considerably lower than that in the *Mpjaz* mutants (around 4,000-fold) (Monte et al., 2019). Besides, compared with those in *Mpjaz*, changes of the relative expression of another two JA marker genes, *MpAOS2* and *MpAOC*, were much attenuated in *Mpnmcp* (Supplementary Figure 3). These data suggest a partially activated JA pathway in the *Mpnmcp* mutants. Interestingly, loss-of-function of *AtCRWN1* also results in activated JA pathway in *Arabidopsis* (Jarad et al., 2019). JAs are lipid-derived hormones released as chemical signals required for wounding response, defense against pathogens, and development (Huang et al., 2017). Recent studies show that *Marchantia* utilizes a conserved “JA” signaling module that functions through OPDA rather than JA (Wasternack and Strnad, 2016; Monte et al., 2018). Our results imply that, in the absence of *MpNMCP*, the OPDA may step in to help *Marchantia* defend itself against pathogens. Overall, it appears that the *MpNMCP* have largely retained their function in the sphere of modulating stress response. Alternatively, the *Mpnmcp* mutant transcriptome might largely reflect secondary defects downstream of primary effects from losing *MpNMCP*. To obtain better insights into how this is linked to stress response, one can use an inducible system, for example, estradiol-mediated gene knock-down (Flores-Sandoval et al., 2016), to study immediate downstream events in the nucleus after losing *MpNMCP* activities.

### MpNMCP Indirectly Regulates Perinuclear Location of Chromatin

We also used our functional *MpNMCP:2HA* tagging lines to perform ChIP-seq (chromatin immunoprecipitation coupled to sequencing) to assess if MpNMCP proteins directly participated in perinuclear chromatin anchoring. We speculated that the 6.5- to 7.5-Mb interval region at the V chromosome, which showed preferential localization at the nuclear periphery (Figure 5), would be a good candidate. However, analysis of ChIP-seq libraries did not reveal such enrichment in this genomic interval. Moreover, inconsistent ChIP signals between individual *MpNMCP:2HA* tagging lines were found across the autosomes (data not shown). The ChIP experiments were performed in accordance with our working protocol that we used for MpTCP1 proteins (Karaaslan et al., 2020b), and we verified successful immunoprecipitation of MpNMCP:2HA proteins with Western blot (Supplementary Figure 8). Thus, our data suggest that MpNMCP proteins do not directly interact with chromatin at the nuclear periphery. Still, we cannot fully rule out technical issues generating such negative ChIP results. For instance, MpNMCP proteins might change their conformation or interact with other proteins upon binding to chromatin, leading to epitope masking that prevents immunoprecipitation. Nonetheless, in our view, the negative ChIP-seq results are in favor of a hypothesis that *MpNMCP* is not involved in organizing genome folding at the nuclear periphery like the *Arabidopsis CRWN1*. This notion is supported by our Hi-C data that there was no increase of inter-chromosomal contacts in *Mpnmcp* mutant nuclei (Figure 6B and Supplementary Figure 6), which were otherwise observed in mutants having deficiency in perinuclear chromatin tethering (Hu et al., 2019; Chang et al., 2020). Nevertheless, it is clear that MpNMCP is required for proper chromatin organization, at least for the repeat region at the V chromosome, since it detached from the nuclear periphery and became decondensed in *Mpnmcp* mutants (Figure 5). Such regulation at limited genomic regions is likely to be indirect. The conformational changes of this V chromosome region might be a secondary consequence of losing MpNMCP, for example, due to the thallus growth retardation and the alteration of transcriptome in *Mpnmcp* mutants.

## Materials and Methods

### Plant Material and Plasmid Construction

*Marchantia polymorpha* Tak-1 (male accession) was used as WT (Ishizaki et al., 2008). Plants were cultivated on half-strength Gamborg’s B5 medium containing 1% agar. For growth analysis, gemmae were grown in a controlled environment room at 23°C in long-day conditions (8-h dark and 16-h light) under white light unless specified otherwise.

CRISPR/Cas9 mutations were generated as previously described in Sugano et al. (2014) using *pMpGE_En03*, *pMpGE010* for gRNA1, and *pMpGE011* for gRNA2 (Addgene entry nos. 71535, 71536, and 71537). A double CRISPR/Cas9 approach was carried out to delete a 3-kb genomic fragment at the *MpNMCP* locus, which included most of its protein-coding region (Figure 1). In this regard, two guide RNAs (sgRNA1, 5′-GCAGG CATGCAGAGTCCGAGAGG-3′, and sgRNA2, 5′-TCACACCG AAGACACCACCAGGG-3′; Supplementary Table 2) were generated. Transformation of spores with assembled vector containing the CRISPR/Cas9 and the two sgRNA expression cassettes was performed by following the protocol described in Kubota et al. (2013). T0 plants were selected on plates containing 100 μg/ml of cefotaxime, 10 μg/ml of hygromycin, and 0.5 μM of chlorsulfuron; individual candidate plants with deletion at the *MpNMCP* locus were identified by genotyping PCR.

To generate the *MpNMCP:2HA* complementation construct, the *MpNMCP* CDS was amplified using Tak-1 cDNA as a template with oligos 5′-TGTACAAAAAAGCAGGCTTTACT GCTAAGTGCTGTGAGAGAA-3′ and 5′-CTTTGTACAAGA AAGCTGGGTTACGTGATTAAGAAGTCCCAG-3′; its 2-kb upstream region was amplified by using Tak-1 genomic DNA with oligos 5′-TGTACAAAAAAGCAGGCTTTAGACCT GAATGTAAACCACCG-3′ and 5′-GTGCCACTTCTCTCAC AGCACTTAGCAGTCTTTCTTTCTA-3′. Next, the amplified *MpNMCP* coding sequence was mutagenized with the following two pairs of oligos: first, 5′-GAGCCTGAGACGTACGGACG GACTAGCTTCTGCACGGATAGCTTG-3′ and 5′-CGTCCGT ACGTCTCAGGCTCAGGAAGGTGCAGGAGGCGGA-3′, and second, 5′-AGGGTATCCAGCATAATCTGGTACGTCGTA TGGGTATCCTTCTTTCCCAGAATCTCTCAC-3′ and 5′-GA TTATGCTGGATACCCTTACGACGTACCAGATTACGCTCAG TGTGGCTTCTGTGGTGGTGGGCCAGGTACTAGTACTG-3′. Each pair of these mutagenesis oligos introduced silent mutations to the *MpNMCP* coding region to prevent it from being recognized by sgRNAs. The second pair of oligos also inserted a 2HA tag to create the MpNMCP:2HA fusion protein. Finally, the resulting expression cassette was further subcloned into *pMpGWB301* (Ishizaki et al., 2015) and was used to rescue *Mpnmcp* mutants.

To generate *MpNMCP Pro:*GUS construct, *MpNMCP* promoter (2-kb upstream of gene model) was amplified using Tak-1 genomic DNA with oligos 5′-TGTAC AAAA AAGCA GGCTT TAGA CCTGA ATGT AAAC CACCG-3′ and 5′-GTGCCAC TTCTC TCACAG CACTT AG CAGT CTTTC TTT CTA-3′; the resulting PCR product was subcloned into *pMpGWB224* (Ishizaki et al., 2015).

### Glucuronidase Staining

GUS assays of *MpNMCP Pro:GUS* transgenic thalli were performed as described previously (Althoff et al., 2014). Plants were fixed with 90% ice-cold acetone for 20 min. Fixed plants were immersed in GUS staining solution (100 mM of potassium ferrocyanide, 100 mM of potassium ferricyanide, and 1 mM of X-Gluc), placed under vacuum for 5 min, incubated for 3 h at 37°C, and cleared with ethanol.

### Fluorescence *in situ* Hybridization

The respective repeat of V chromosome (Okada et al., 2001) was amplified from genomic DNA of Tak-1, with oligos 5′-GGATCCGGATGCCGAATATAT-3′ and 5′-GGATCCGCGAAACTTCTGGG-3′ (Supplementary Table 2). To label probes, PCR amplification was carried out using DNA polymerase with 50% of dTTP replaced with digoxigenin-11-dUTP (Product Number 3359247910, Roche, United States) according to the manufacturer’s instructions. The test of probe male-specificity was done by chromosome FISH and was detected with Alexa Fluor 488 (Green) (Abcam, Cat# ab150077).

Preparation of chromosome spreads from young thalli of *Marchantia* and FISH were performed following a standard procedure with minor modifications (Nakayama et al., 2001; Okada et al., 2001; Yamato et al., 2007). In brief, for chromosome preparation, 2-week-old thalli were fixed by a fixative (ethanol:acetic acid 3:1) at 4°C for 30 min. Then, the fixative was replaced with 70% ethanol and stored at 4°C overnight. Fixed thalli were washed with de-ionized water, followed by the enzyme maceration at 37°C for 1 h. After being rinsed, each piece of thallus was laid onto a clean glass slide, and cells were spread by an easy “SteamDrop” method (Kirov et al., 2014). The next FISH steps were performed exactly as described in the method part of Okada et al. (2000). Briefly, 25 ng digoxigenin (DIG)-labeled V chromosome targeting probe was further hybridized with preparations in 20 ml of a hybridization mixture (Bi et al., 2017). The mixture was denatured at 85°C for 10 min and transferred to preparations. Then, the preparations were heated at 85°C for 30 min and hybridized in a hybridizer (Agilent, United States) at 37°C overnight. For FISH probe detection, Monoclonal Anti-Digoxin (Sigma, Catalog # D8156) and Alexa Fluor 488-conjugated goat anti-Mouse IgG (Invitrogen, Catalog # A-11017) were used as the primary and secondary antibodies, respectively.

DNA FISH in *Marchantia* nuclei was adapted from Bi et al. (2017). Around 5,000 nuclei were sorted with a Beckman Coulter MofFlo XDP as described in Karaaslan et al. (2020a). The sorted nuclei were centrifuged for 3,000 × *g* at 4°C for 7 min. The supernatant was discarded after centrifugation, and the pellet was resuspended in 30 μL of 1 × phosphate-buffered saline (PBS). The nuclei were incubated at 65°C for 30 min and mixed with 5 μL of 0.1 mg/ml RNase A. A circle (∼1 cm^2^) was drawn on the Superfrost Ultra Plus Adhesion Slide (Thermo Fisher Scientific) using a DAKO pen to avoid deforming the specimen once mounted with a coverslip. All the suspensions were transferred onto the slide and incubated in a moist chamber at 37°C. At the end of RNase A treatment, the nuclei became attached to the glass slide. Next, the slide was rinsed in 2 × SSC and dehydrated in a graded series of alcohol solutions at room temperature (RT). Twenty microliters of the probe mixture (same contents as in chromosome FISH) was denatured at 95°C for 10 min and transferred immediately to ice. Twenty microliters of denatured probe was added to the slide and hybridized in a moist chamber at 37°C overnight. All subsequent steps, including washing and detection, were strictly followed (Bi et al., 2017).

### Immunohistostaining

Around 5,000 sorted nuclei from 1% formaldehyde fixed Tak-1 or *MpNMCP Pro:MpNMCP-2HA* seedlings were used. After the nuclei were treated with RNase A at 37°C for 1 h, the nuclei were washed three times with PBS. Then, an antigen retrieval step was performed with Universal HIER antigen retrieval reagent (Abcam) by following manufacturer’s instructions. The nuclei were then incubated in blocking solution [5% bovine serum albumin (BSA) in 0.2% Tween 20 4 × SSC] and then mounted unto glass microscopy slides. Slides were incubated with HA Tag Alexa Fluor 647 conjugate (Thermo Fisher Scientific) at 1:500 diluted with blocking solution at 37°C for 1 h. After three washes with PBS, slides were mounted with SlowFade Diamond Antifade Mountant, to which 4′,6-diamidino-2-phenylindole (DAPI) (Sigma-Aldrich, St. Louis, United States) was added to visualize nuclei (Sigma-Aldrich, St. Louis, United States).

### Fluorescence Microscopy and Image Processing

All confocal image acquisition of nuclei were performed on an LSM 880 confocal laser scanning microscope (Zeiss) with Airyscan system equipped with a 63 × /1.4 numerical aperture (NA) water objective. For the processing of acquired images, ImageJ (NIH) was used to pseudocolor and merge.

### *In situ* Hi-C and Data Processing

The *in situ* Hi-C library preparation was strictly followed as described (Karaaslan et al., 2020b). In total, two replicates of 2-week-old Tak-1 and *Mpnmcp* mutant thallus Hi-C libraries were made, and for each replicate, around 0.5 g of fixed sample was homogenized for nuclei isolation. The libraries were sequenced on an Illumina HiSeq 3000 instrument with 2 × 150 bp reads. Hi-C reads mapping, filtering, and Hi-C map normalization were done according to our recent Hi-C study (Karaaslan et al., 2020b). The bin size setting for genome-wide and individual chromosome Hi-C maps was 50 and 10 kb, respectively.

### RNA-seq Library Preparation and Analysis

RNA-seq was performed with three biological replicates per sample. Total RNA was extracted from 23-day-old *Mpnmcp* mutants or Tak-1 thalli using an RNeasy Plant Mini Kit (Qiagen). After treatment with DNase I (Thermo Scientific, Waltham, MA, United States), the RNA was mixed with first-strand buffer (Invitrogen), heated at 80°C for 2 min followed by 94°C for 1.5 min. Then the Poly A^+^ RNA was purified from the total RNA and processed strictly according to manufacturer’s recommendations. After performing end repair, the free end of the newly synthesized double-stranded cDNA was ligated to the reverse adaptor, and final enrichment PCR was performed to generate libraries for sequencing.

RNA-seq reads were aligned against the Tak-1 v5 genome using TopHat 2 (v2.1.1). DEGs were identified with the DESeq2 package in R. The criteria for calling DEGs was set as false discovery rate smaller than 0.01 and expression fold change more than 3. Details of the reads count table and DEGs can be found in Supplementary Table 1.

## Data Availability Statement

The datasets presented in this study can be found in online repositories. The names of the repository/repositories and accession number(s) can be found below: https://www.ncbi.nlm.nih.gov/, PRJNA692743. Large datasets including Hi-C matrices (10 kb bin size for individual chromosomes, 50 kb bin size for the whole genome) are accessible with the following link: https://figshare.com/articles/dataset/Characterization_of_a_plant_lamin-like_protein_in_Marchantia/13568276. The following public RNA-seq datasets were downloaded and re-analyzed as for those generated in this study: Phytophthora palmivora infection (PRJNA397637, https://www.ncbi.nlm.nih.gov/sra), and salt stress (PRJDB6783, https://ddbj.nig.ac.jp). DEGs from in the Mpjaz mutants, which were based on a microarray experiment, were from Monte et al. (2019); we converted their gene annotation to the Tak1 v5 format with an online tool at https://marchantia.info/utils/id_converter/.

## Author Contributions

CL conceived this study. CL and NW designed the experiments. NW performed most of the experiments. NW, EK, and NF established and characterized transgenic lines. KB performed nuclei sorting. CL and NW prepared the manuscript. All authors contributed to the article and approved the submitted version.

## Conflict of Interest

The authors declare that the research was conducted in the absence of any commercial or financial relationships that could be construed as a potential conflict of interest.

## Supplementary Material

Supplementary Figure 1 Sequence alignment of NMCP homologs.

Supplementary Figure 2 RNA-seq analysis of *Mpnmcp* mutant lines.

Supplementary Figure 3 Relative expression of selected jasmonate marker genes.

Supplementary Figure 4 Perinuclear localization of a V chromosome region.

Supplementary Figure 5 FISH experiment of the entire V-chromosome.

Supplementary Figure 6 Genome-wide Hi-C maps of WT Tak-1 and *Mpnmcp* mutant thalli.

Supplementary Figure 7 Comparison of chromatin organization patterns of autosomes in WT Tak-1 and *Mpnmcp* mutants.

Supplementary Figure 8 Western blot confirms MpNMCP-2HA protein IP.

Supplementary Figure 9 Fluorescence-activated cell sorting (FACS) of 8C nuclei.

Supplementary Figure 10 Thalli area measurements of WT Tak-1, *Mpnmcp* mutants and *Mpnmcp* complementation lines.

Supplementary Figure 11 Semi-quantitative RT-PCR showing the expression of *MpNMCP* in the *crwn1 crwn2 35S:MpNMCP-2HA* plants.

Supplementary Table 1 Read count and DEGs tables.

Supplementary Table 2 Sequences of primer pairs used in this study.

Supplementary Data Set 1 The NMCP sequences used to reconstruct Supplementary Figure 1.
