# Supplementary material for: Characterization of a Plant Nuclear Matrix Constituent Protein in Liverwort
Source: Front Plant Sci. 2021 May 7;12:670306. doi: 10.3389/fpls.2021.670306 (PMC8139558; doi:10.3389/fpls.2021.670306)
Supplement: Supplementary Figure 1 — Sequence alignment of NMCP homologs. [file Data_Sheet_2.DOCX]

**Supplemental information**

**Characterization of a plant nuclear matrix constituent protein in liverwort**

**Nan Wang,** **Ezgi Süheyla Karaaslan, Natalie Faiss, Kenneth Wayne Berendzen and Chang Liu**


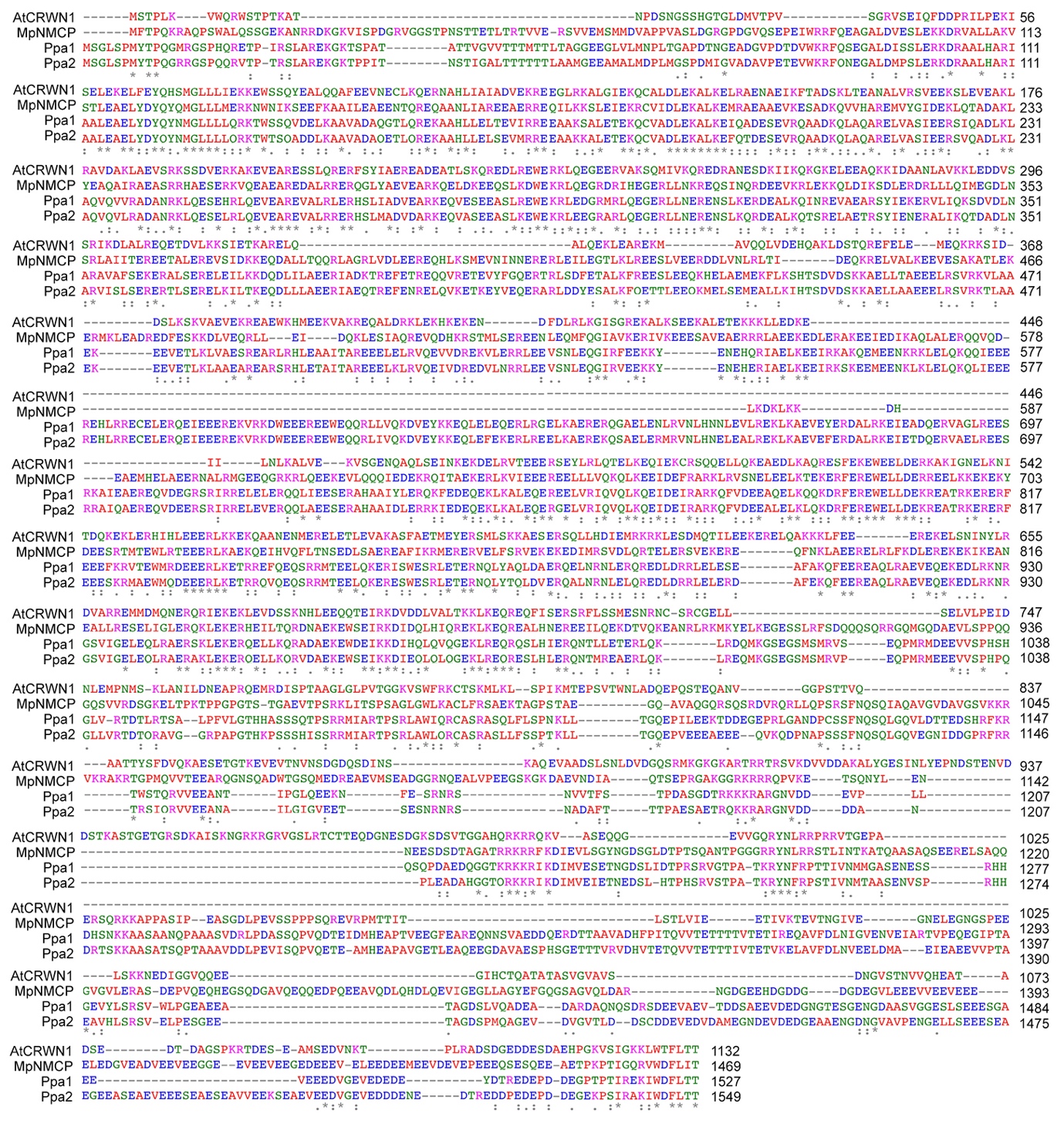


**Supplementary Figure 1. Sequence alignment of NMCP homologs.**

AtCRWN1 denotes *Arabidopsis thaliana* CRWN1, Ppa1 and Ppa2 denote the two *Physcomitrella patens* NMCPs, and MpNMCP denotes *Marchantia polymorpha* NMCP. Homology analyses were conducted with EMBL tools (<https://www.ebi.ac.uk/Tools/msa/mafft/>). The sequences used to reconstruct the multiple sequence alignment are provided in Supplementary Data Set 1.


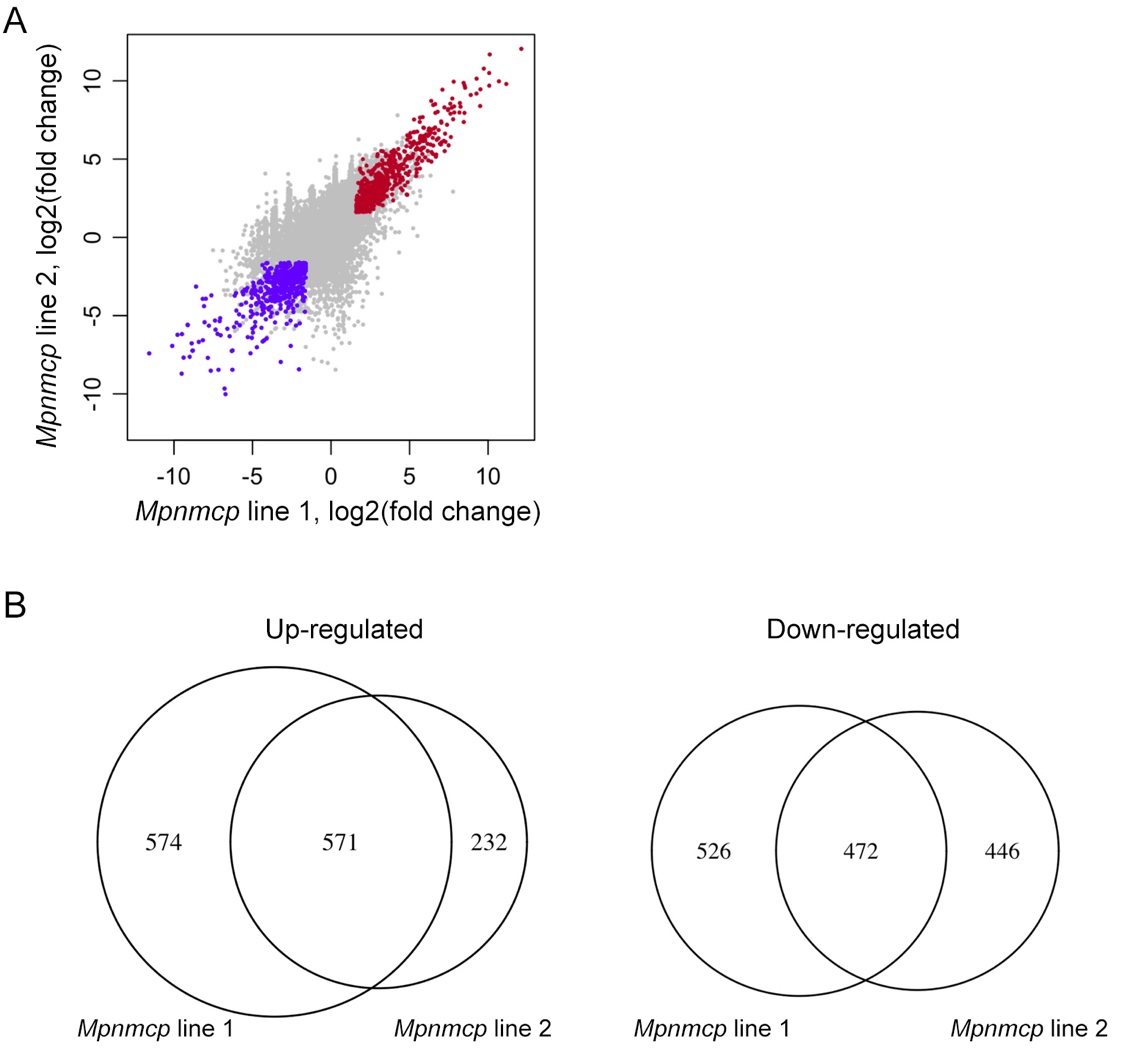


**Supplementary Figure 2. RNA-seq analysis of *Mpnmcp* mutant lines.**

(A) Scatter plot of gene expression fold change. Red and purple datapoints indicate Up- and Down-regulated genes shared between the two mutant lines, respectively. (B) Venn diagram of differentially expressed genes.

**
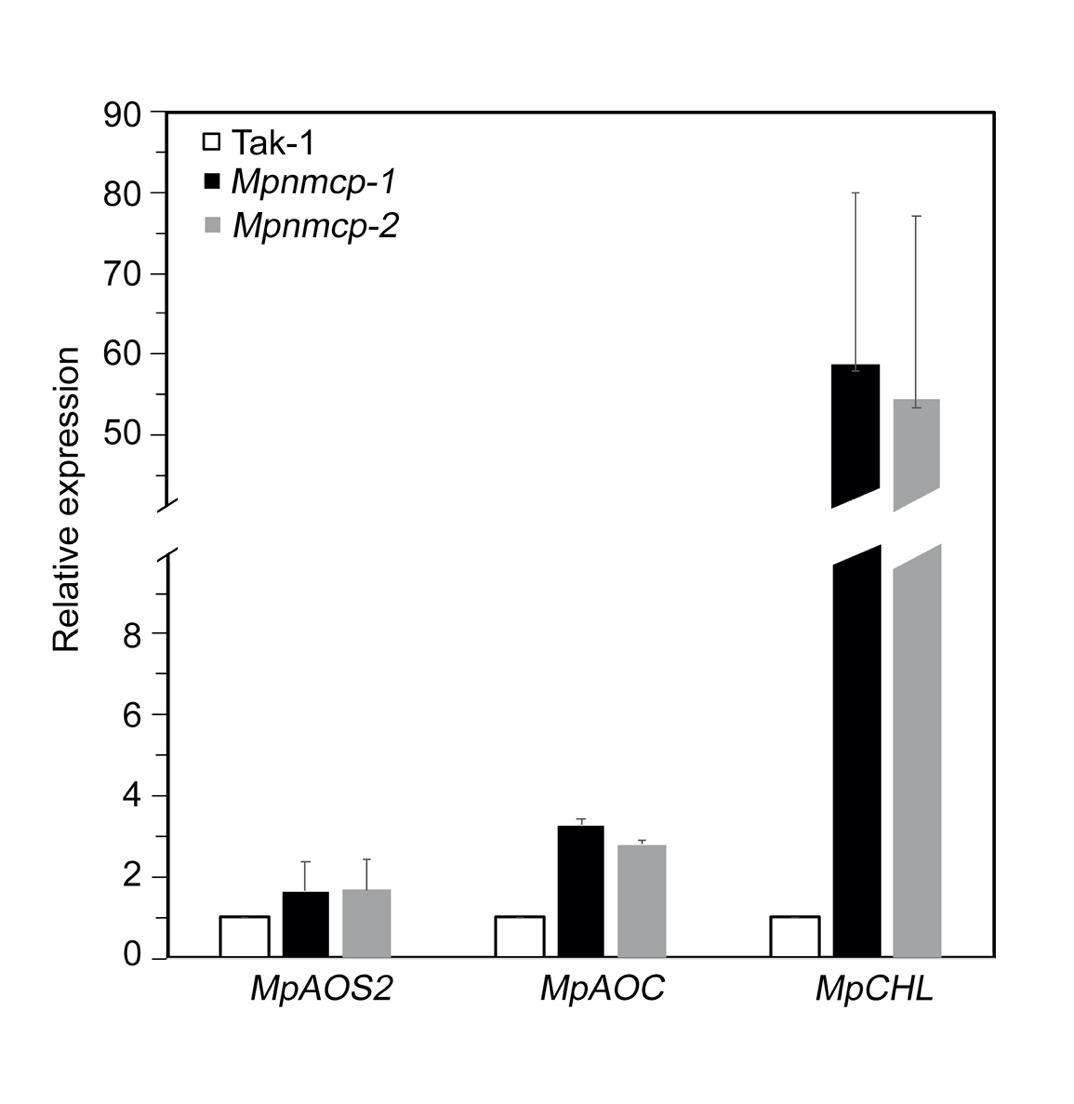
**

**Supplementary Figure 3. Relative expression of selected jasmonate marker genes**.

Relative expression was normalized to an *ACTIN* control. Primer pairs for RT-PCR detection are as previously described (Monte et al., 2019) (Primers were also listed in Table S2).


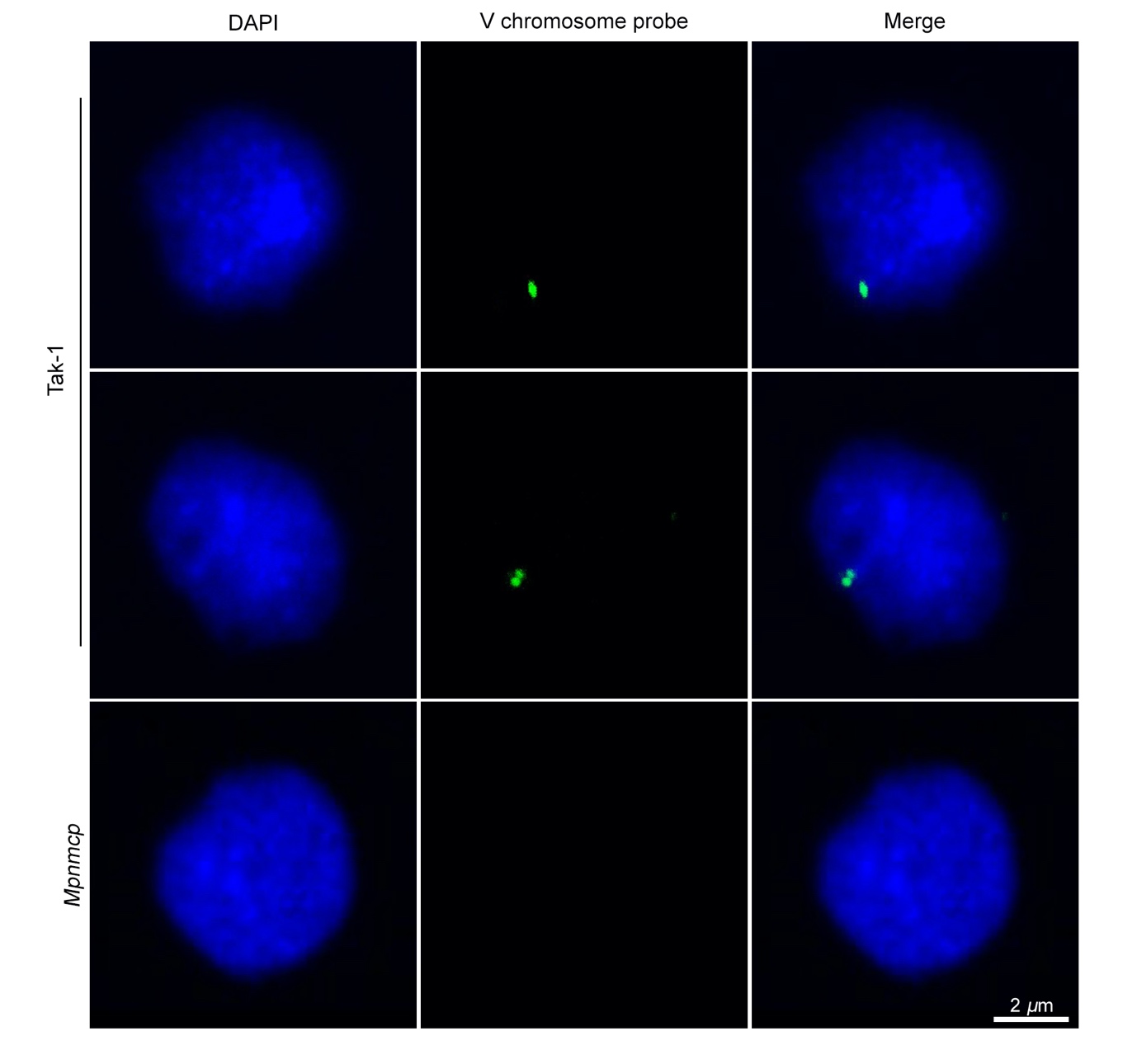
 **Supplementary Figure 4. Perinuclear localization of a V chromosome region.**

Representative FISH images showing localization of a selected repeat genomic region at V chromosome in Tak-1 (upper and middle panels) and *Mpnmcp mutant* (bottom panel). Nuclei were isolated from vegetative thalli fixed with 1% formaldehyde. Note that FISH signal is not detectable in the *Mpnmcp* nucleus. Scale bar shows 2 *µ*m.

**
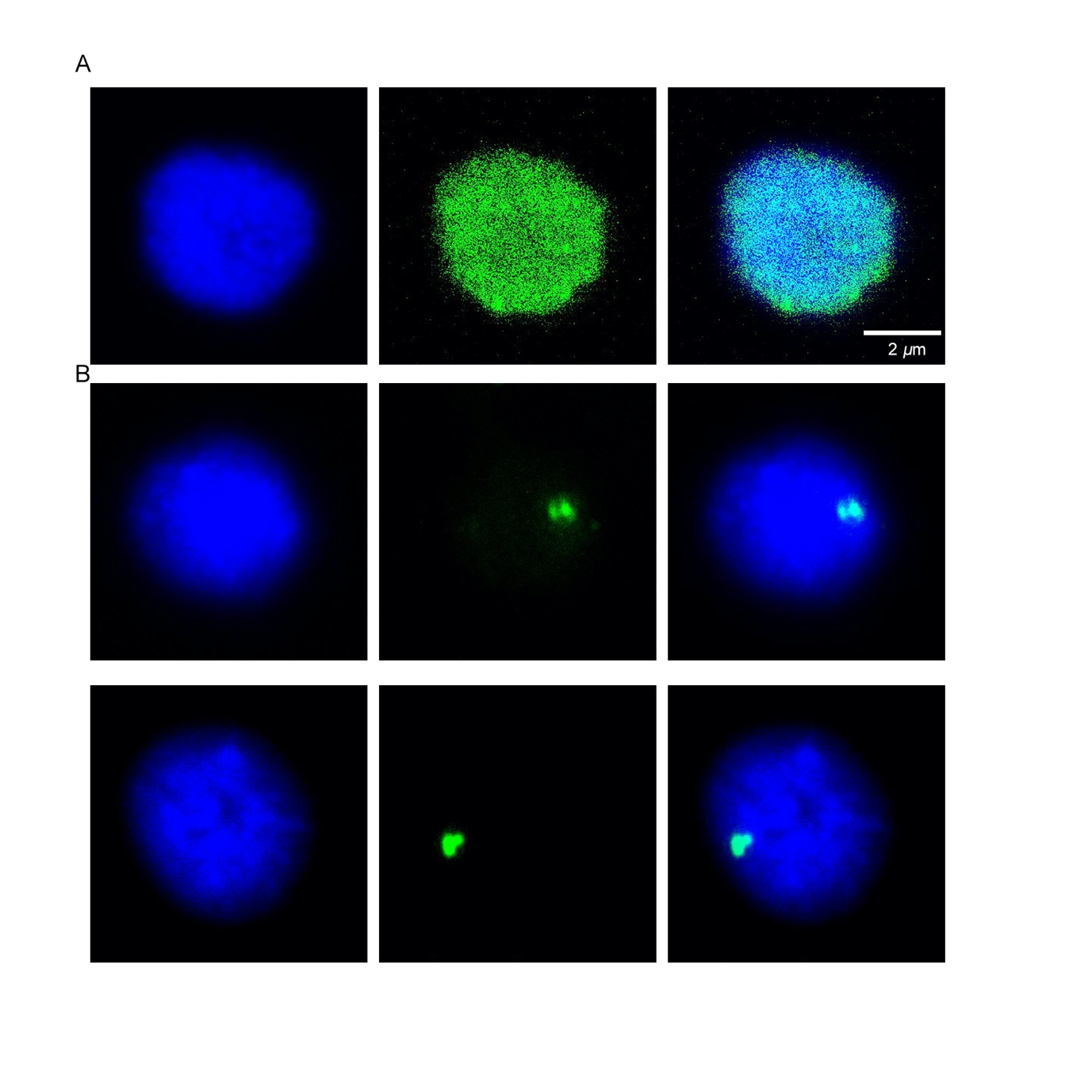
**

**Supplementary Figure 5. FISH experiment of the entire V-chromosome.**

(A) FISH using labeled Tak-1 genomic DNA as probes. (B) FISH using labeled Tak-1 genome DNA mixed with 5 times excessive unlabeled Tak-2 genomic DNA as probes. Nuclei were stained with DAPI to visualize DNA (blue channel). The green channel shows probe signals. Scale bar denotes 2 *µ*m.


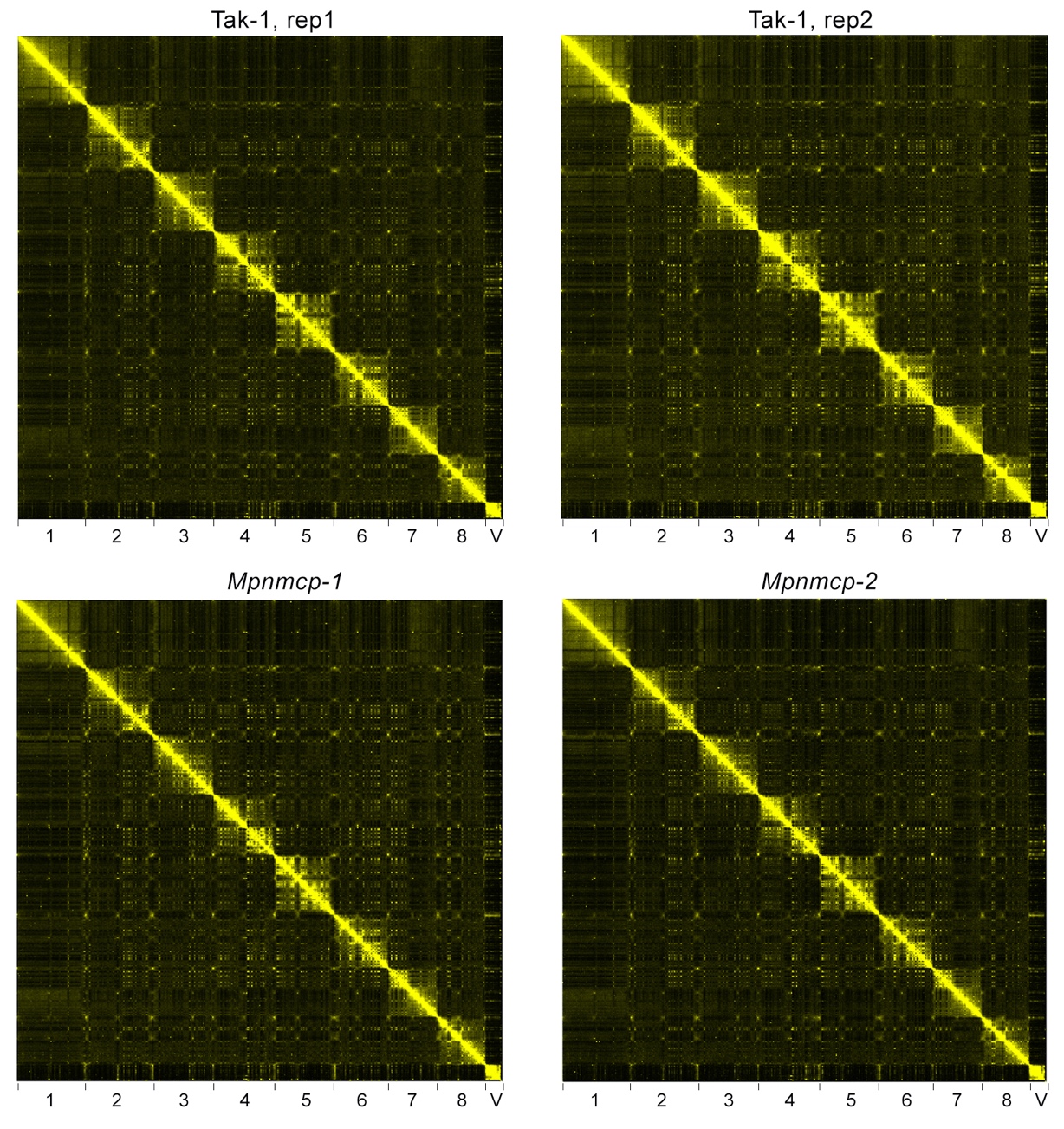


**Supplementary Figure 6. Genome-wide Hi-C maps of WT Tak-1 and *Mpnmcp* mutant thalli.**

Each Hi-C map was normalized with 50 kb bins.


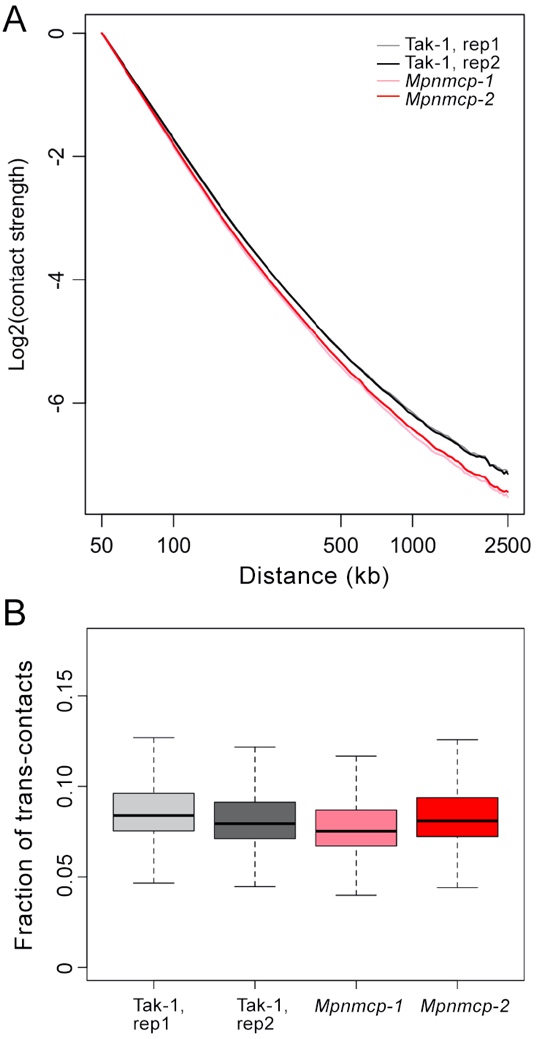


**Supplementary Figure 7. Comparison of chromatin organization patterns of autosomes in WT Tak-1 and *Mpnmcp* mutants.**

(A) Comparison of interaction decay components. Each curve represents the average of the 8 autosomes in a genotype. (B) Boxplots showing fraction of inter-chromosomal contacts of individual 50 kb regions in autosomes.

**
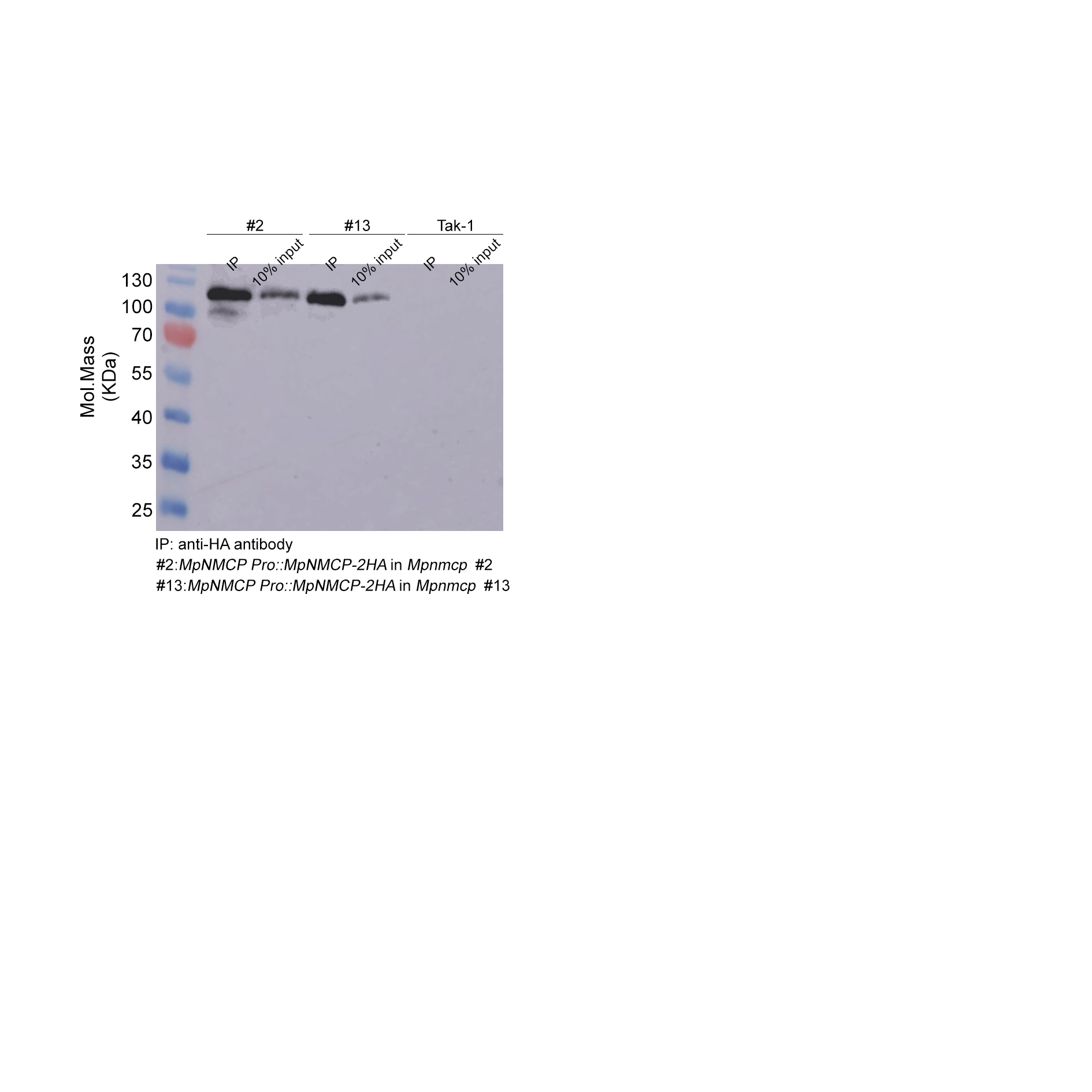
**

**Supplementary Figure 8. Western blot confirms MpNMCP-2HA protein IP.**

Tak-1 and *MpNMCP Pro::MpNMCP-2HA* (in *Mpnmcp*) tagging lines, which are labeled as #2 and #13, were subjected to ChIP using anti-HA beads for IP . “Input” stands for the supernatant obtained after chromatin sonication; “IP” stands for proteins immunoprecipitated with anti-HA beads. Anti-HA HRP (Horseradish Peroxidase) conjugate was used for detecting proteins on the membrane.

**
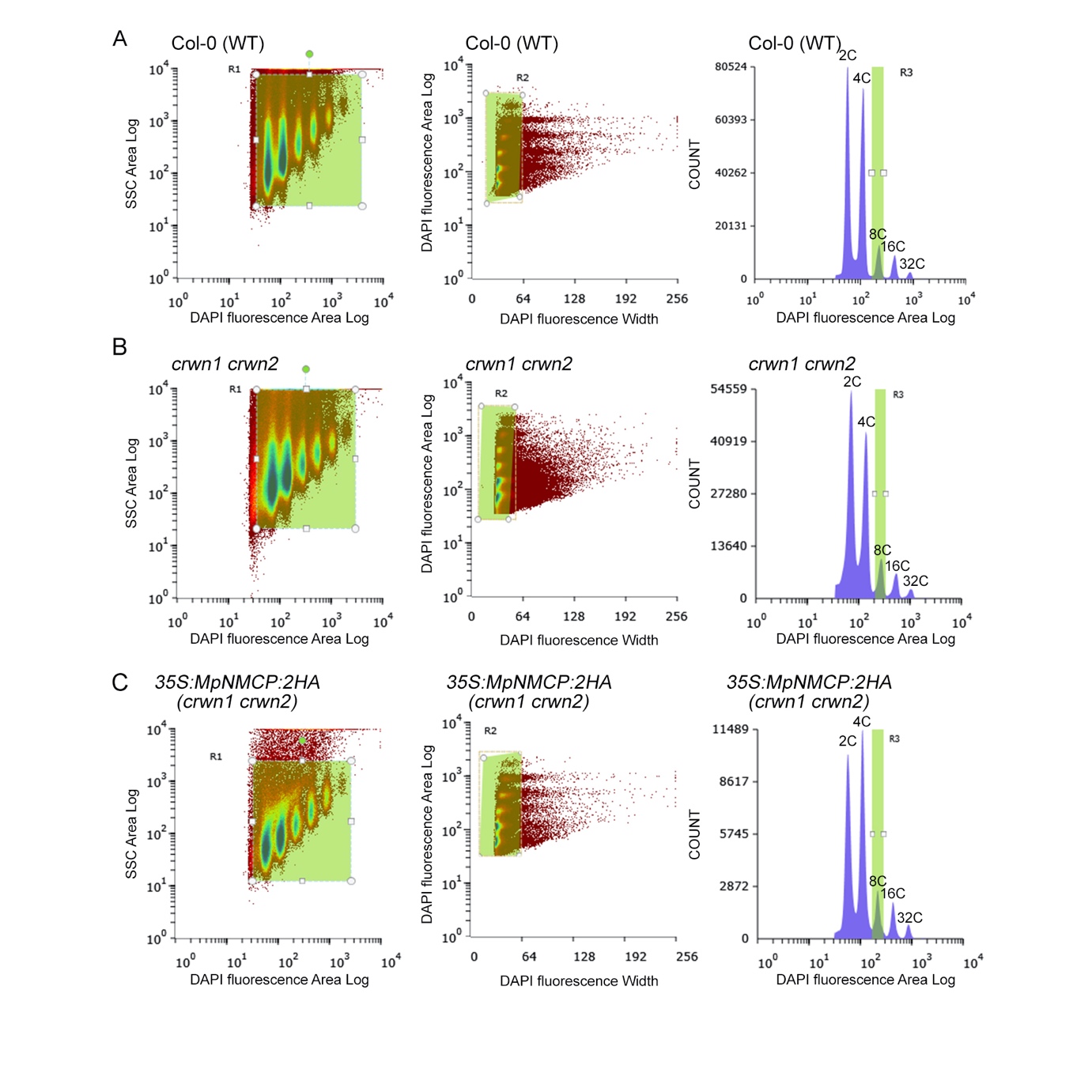
**

**Supplementary Figure 9. Fluorescence-activated cell sorting (FACS) of 8C nuclei.**

Representative flow cytometric identification of endoreduplication nuclei using S3e Cell Sorter (Bio-Rad). Gating strategy of Col-0 (WT) (A), *crwn1 crwn2* (B) and *crwn1 crwn2 35S:MpNMCP:2HA* (C) with 2C, 4C, 8C, 16C, and 32C nuclei. DNA content was assessed according to DAPI fluorescent intensity. DAPI was excited at 405 nm and its emission captured at 447/60 nm (DAPI channel). To determine nuclei endopolyploidy levels, nuclei were resolved based on the Side Scatter (SSC), DAPI channel pulse width, and DAPI channel pulse area. R1, R2, and R3 in each panel refer to a sequential gating strategy for selecting 8C nuclei, which were subsequently examined under the microscope (Figure 4A).


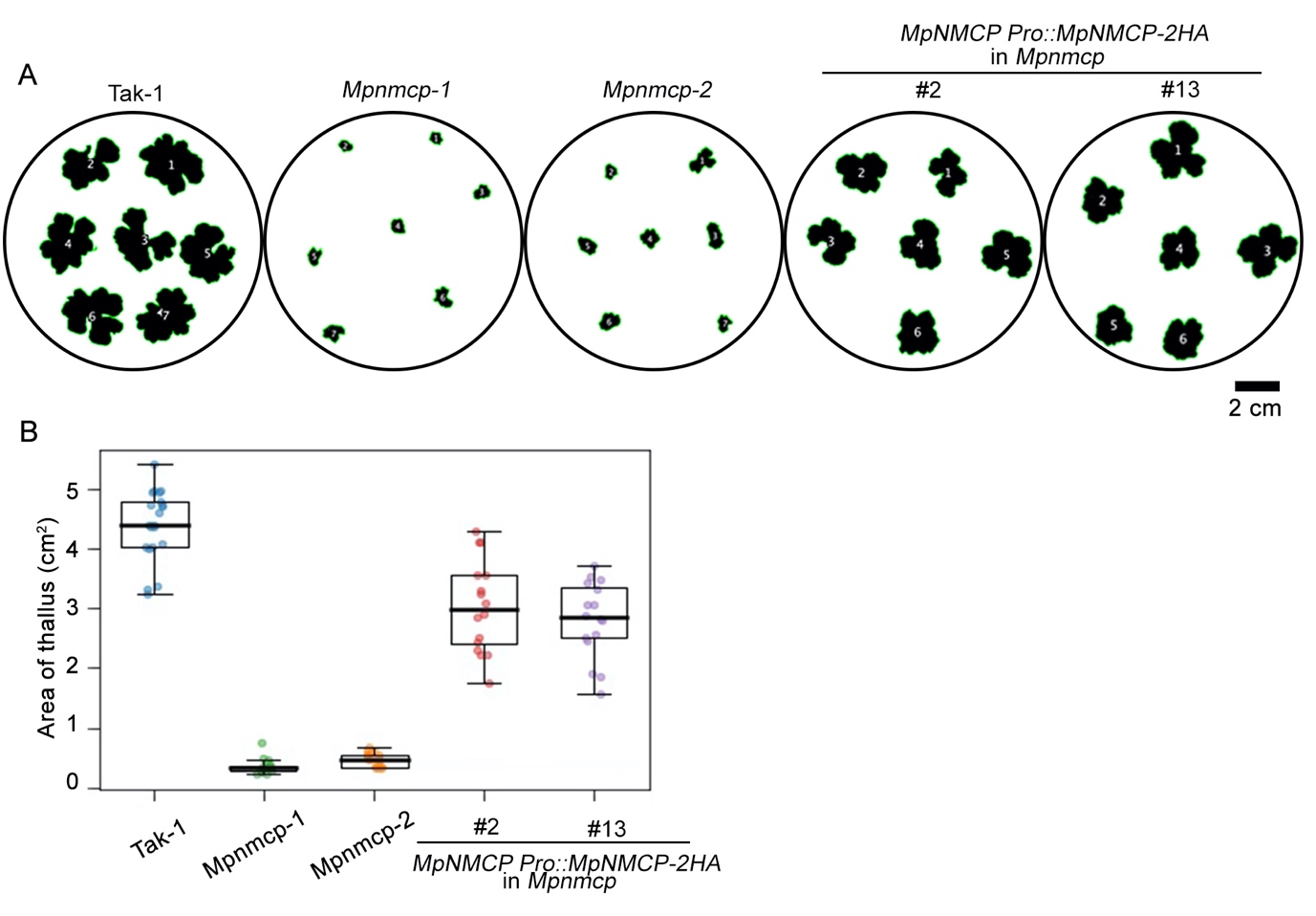


**Supplementary Figure 10. Thalli area measurements of WT Tak-1, *Mpnmcp* mutants and *Mpnmcp* complementation lines.**

(A) Silhouettes of plants shown in Figure 1D. The optical surface areas of thalli were measured by ImageJ. Scale bar = 2 cm. (B) Area of thalli (n ≥ 16 plants).


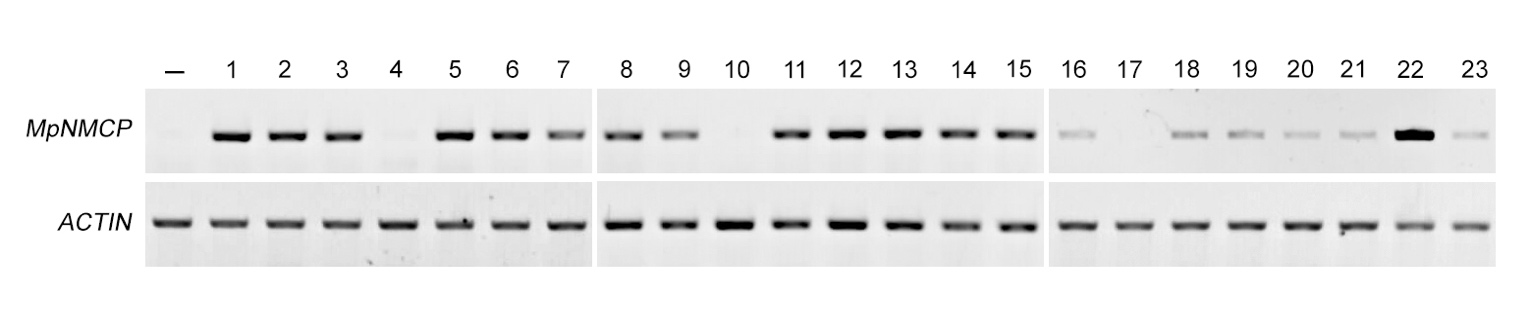


**Supplementary Figure 11. Semi-quantitative RT-PCR showing the expression of *MpNMCP* in the *crwn1 crwn2 35S:MpNMCP-2HA* plants.**

The figure shows for each sample 5 ul of the same amplification reaction after 24 PCR cycles. *ACTIN* was used as the reference gene. A negative control sample that does not contain the *35S:MpNMCP-2HA* transgene is indicated with “-”.

**Reference:**

Monte, I., Franco-Zorrilla, J.M., Garcia-Casado, G., Zamarreno, A.M., Garcia-Mina, J.M., Nishihama, R., et al. (2019). A Single JAZ Repressor Controls the Jasmonate Pathway in Marchantia polymorpha. *Mol Plant*. 12, 185-198.doi:10.1016/j.molp.2018.12.017
