# Supplementary material for: Characterization of a Plant Nuclear Matrix Constituent Protein in Liverwort
Source: Front Plant Sci. 2021 May 7;12:670306. doi: 10.3389/fpls.2021.670306 (PMC8139558; doi:10.3389/fpls.2021.670306)
Supplement: Supplementary Data Set 1 — The NMCP sequences used to reconstruct Supplementary Figure 1. [file Data_Sheet_1.DOCX]

<https://www.uniprot.org/uniprot/F4HRT5>

**>CRWN1 Arabidopsis thaliana AT1G67230. 1**

MSTPLKVWQRWSTPTKATNPDSNGSSHGTGLDMVTPVSGRVSEIQFDDPRILPEKISELEKELFEYQHSMGLLLIEKKEWSSQYEALQQAFEEVNECLKQERNAHLIAIADVEKREEGLRKALGIEKQCALDLEKALKELRAENAEIKFTADSKLTEANALVRSVEEKSLEVEAKLRAVDAKLAEVSRKSSDVERKAKEVEARESSLQRERFSYIAEREADEATLSKQREDLREWERKLQEGEERVAKSQMIVKQREDRANESDKIIKQKGKELEEAQKKIDAANLAVKKLEDDVSSRIKDLALREQETDVLKKSIETKARELQALQEKLEAREKMAVQQLVDEHQAKLDSTQREFELEMEQKRKSIDDSLKSKVAEVEKREAEWKHMEEKVAKREQALDRKLEKHKEKENDFDLRLKGISGREKALKSEEKALETEKKKLLEDKEIILNLKALVEKVSGENQAQLSEINKEKDELRVTEEERSEYLRLQTELKEQIEKCRSQQELLQKEAEDLKAQRESFEKEWEELDERKAKIGNELKNITDQKEKLERHIHLEEERLKKEKQAANENMERELETLEVAKASFAETMEYERSMLSKKAESERSQLLHDIEMRKRKLESDMQTILEEKERELQAKKKLFEEEREKELSNINYLRDVARREMMDMQNERQRIEKEKLEVDSSKNHLEEQQTEIRKDVDDLVALTKKLKEQREQFISERSRFLSSMESNRNCSRCGELLSELVLPEIDNLEMPNMSKLANILDNEAPRQEMRDISPTAAGLGLPVTGGKVSWFRKCTSKMLKLSPIKMTEPSVTWNLADQEPQSTEQANVGGPSTTVQAATTYSFDVQKAESETGTKEVEVTNVNSDGDQSDINSKAQEVAADSLSNLDVDGQSRMKGKGKARTRRTRSVKDVVDDAKALYGESINLYEPNDSTENVDDSTKASTGETGRSDKAISKNGRKRGRVGSLRTCTTEQDGNESDGKSDSVTGGAHQRKRRQKVASEQQGEVVGQRYNLRRPRRVTGEPALSKKNEDIGGVQQEEGIHCTQATATASVGVAVSDNGVSTNVVQHEATADSEDTDAGSPKRTDESEAMSEDVNKTPLRADSDGEDDESDAEHPGKVSIGKKLWTFLTT

<https://www.uniprot.org/uniprot/A0A2K1L6J7>

**>Ppa1 Physcomitrella patens Phytozome 12 Pp3c2_3483 0V3.1 Alias Pp1s76_81V 6**

MSGLSPMYTPQGMRGSPHQRETPIRSLAREKGKTSPATATTVGVVTTTMTTLTAGGEEGLVLMNPLTGAPDTNGEADGVPDTDVWKRFQSEGALDISSLERKDRAALHARIAALEAELYDYQYNMGLLLLQRKTWSSQVDELKAAVADAQGTLQREKAAHLLELTEVIRREEAAKSALETEKQCVADLEKALKEIQADESEVRQAADKQLAQARELVASIEERSIQADLKLAQVQVVRADANRKLQESEHRLQEVEAREVALRLERHSLIADVEARKEQVESEEASLREWEKRLEDGRMRLQEGERLLNERENSLKERDEALKQINREVAEARSYIEKERVLIQKSDVDLNARAVAFSEKERALSERELEILKKDQDLILAEERIADKTREFETREQQVRETEVYFGQERTRLSDFETALKFREESLEEQKHELAEMEKFLKSHTSDVDSKKAELLTAEEELRSVRKVLAAEKEEVETLKLVAESREARLRHLEAAITAREEELELRVQEVVDREKVLERRLEEVSNLEQGIRFEEKKY ENEHQRIAELKEEIRKAKQEMEENKRKLELQKQQIEEEREHLRRECELER QEIEEEREKVRKDWEEEREEWEQQRLLVQKDVEYKKEQLELEQERLRGELKAERERQGAELENLRVNLHNNLEVLREKLKAEVEYERDALRKEIEADQERVAGLREESRKAIEAEREQVDEGRSRIRRELELERQQLIEESERAHAAIYLERQKFEDEQEKLKALEQEREELVRIQVQLKQEIDEIRARKQFVDEEAQELKQQKDRFEREWELLDEKREATRKERERFEEEFKRVTEWMRDEEER LKETRREFQEQSRRMTEELQKERISWESRLETERNQLYAQLDAERQEL NRNLERQREDLDRRLELESEAFAKQFEEREAQLRAEVEQEKEDLRKNRGSVIGELEQLRAERSKLEKERQELLKQRADAEKEWDEIKKDIHQLQVQGEKLREQRQSLHIERQNTLLETERLQKLRDQMKGSEGSMSMRVSEQPMRMDEEVVSPHSHGLVRTDTLRTSALPFVLGTHHASSSQTPSRRMIARTPSRLAWIQRCASRASQLFLSPNKLLTGQEPILEEKTDDEGEPRLGANDPCSSFNQSQLGQVLDTTEDSHRFKRTWSTQRVVEEANTIPGLQEEKNFESRNRSNVVTFSTPDASGDTRKKKRARGNVDDEVPLLQSQPDAEDQGGTKRKKRIKDIMVESETNGDSLIDTPRSRVGTPATKRYNFRPTTIVNMMGASENESSRHHDHSNKKAASAANQPAAASVDRLPDASSQPVQDTEIDMHEAPTVEEGFEAREQNNSVAEDDQERDTTAAVADHFPITQVVTETTTTVTETIREQAVFDLNIGVENVEIARTVPEQEGIPTAGEVYLSRSVWLPGEAEEATAGDSLVQADEADARDAQNQSDRSDEEVAEVTDDSAEEVDEDGNGTESGENGDAASVGGESLSEEESGAEEVEEEDVGEVEDEDEYDTRE DEPDDEGPTPTIREKIWDFLTT

**>Ppa2 Physcomitrella patens Phytozome 12 Pp3c1_1360 V3.1 Alias Pp1s200_64 V6.1**

MSGLSPMYTPQGRRGSPQQRVTPTRSLAREKGKTPPITNSTIGALTTTTTTLAAMGEEAMALMDPLMGSPDMIGVADAVPETEVWKRFQNEGALDMPSLERKDRAALHARIAALEAELYDYQYNMGLLLLQRKTWTSQADDLKAAVADAQETLQREKAAHLLELSEVMRREEAAKKALETEKQCVADLEKALKEFQTDESEVRQAADKQLAQARELVASIEERSVQADLKLAQVQVLRADANRKLQESELRLQEVEAREVALRRERHSLMADVDARKEQVASEEASLKEWEKRLEEGRARLQEGERLLNERENSLKQRDEALKQTSRELAETRSYIENERALIKQTDADLNARVISLSERERTLSERELKILTKEQDLLLAEERIAEQTREFENRELQVKETKEYVEQERARLDDYESALKFQETTLEEQKMELSEMEALLKIHTSDVDSKKAELLAAEEELRSVRKTLAAEKEEVETLKLAAEAREARSRHLETAITAREEELKLRVQEIVDREDVLNRRLEEVSNLEQGIRVEEKKYENEHERIAELKEEIRKSKEEMEENKLKLELQKQLIEEEREHLRRECELERQEIEEEREKVRKDWEEEREEWEQQRLIVQKDVEYKKEQLEFEKERLREELKAEREKQSAELERMRVNLHNELEALREKLKAEVEFERDALRKEIETDQERVAELREESRRAIQAEREQVDEERSRIRRELEVERQQLAEESERAHAAIDLERRKIEDEQEKLKALEQERGELVRIQVQLKQEIDEIRARKQFVDEEALELKLQKDRFEREWELLDEKREATRKERERFEEESKRMAEWMQDEEERLKETRRQVQEQSRRMTEELQKERESWESRLETERNQLYTQLDVERQALNRNLELQREDLDRRLELERDAFEKQFEEREAQLRAEVEQEKEDLRKNRGSVIGELEQLRAERAKLEKERQELLKQRVDAEKEWSEIKKDIEQLQLQGEKLREQRESLHLERQNTMREAERLQKLREQMKGSEGSMSMRVPEQPMRMEEEVVSPHPQGLLVRTDTQRAVGGRPAPGTHKPSSSHISSRRMIARTPSRLAWLQRCASRASLLFSSPTKLLTGQEPVEEEAEEEQVKQDP NAPSSSFNQSQLGQVEGNIDDGPRFRRTRSIQRVVEEANAILGIGVEETSESNRNRSNADAFTTTPAESAETRQKKRARGNVDDDDANPLEADAHGGTQRKKRIKDIMVEIETNEDSLHTPHSRVSTPATKRYNFRPSTIVNMTAASENVSPRHHDRTSKKAASATSQPTAAAVDDLPEVISQPVQETEAMHEAPAVGETLEAQEEGDAVAESPHSGETTTVRVDHVTETQVVTETTTIVTETVKELAVFDLNVEELDMAEIEAEEVVPTAEAVHLSRSVELPESGEETAGDSPMQAGEVDVGVTLDDSCDDEVEDVDAMEGNDEVDEDGEAAENGDNGVAVPENGELLSEEESEAEGEEASEAEVEEESEAESEAVVEEKSEAEVEEDVGEVEDDDENEDTREDDPEDEPDDEGEKPSIRAKIWDFLTT

<https://www.uniprot.org/uniprot/A0A2R6W3X2>

>Mapoly0160s0011.1 MpNMCP
MFTPQKRAQPSWALQSSGEKANRRDKGKVISPDGRVGGSTPNSTTETLTRTVVERSVVEMSMMDVAPPVASLDGRGPDGVQSEPEIWRRFQEAGALDVESLEKKDRVALLAKVSTLEAELYDYQYQMGLLLMERKNWNIKSEEFKAAILEAEENTQREQAANLIAREEAERREQILKKSLEIEKRCVIDLEKALKEMRAEAAEVKESADKQVVHAREMVYGIDEKLQTADAKLYEAQAIRAEASRRHAESERKVQEAEAREDALRRERQGLYAEVEARKQELDKEEQSLKDWEKRLQEGRDRIHEGERLLNKREQSINQRDEEVKRLEKKQLDIKSDLERDRLLLQIMEGDLNSRLAIITEREETALEREVSIDKKEQDALLTQQRLAGRLVDLEEREQHLKSMEVNINNERERLEILEGTLKLREESLVEERDDLVNLRLTIDEQKRELVALKEEVESAKATLEKERMKLEADREDFESKKDLVEQRLLEIDQKLESIAQREVQDHKRSTMLSEREENLEQMFQGIAVKERIVKEEESAVEAERRRLAEEKEDLERAKEEIEDIKAQLALERQQVQDLKDKLKKDHEAEMHELAEERNALRMGEEQGRKRLQEEKEVLQQQIEDEKRQITAEKERLKVIEEEREELLLVQKQLKEEIDEFRARKLRVSNELEELKTEKERFEREWELLDERREELKKEKEKYDEESRTMTEWLRTEEERLKAEKQEIHVQFLTNSEDLSAEREAFIKRMERERVELFSRVEKEKEDIMRSVDLQRTELERSVEKEREQFNKLAEERELRLFKDLEREKEKIKEANEALLRESELIGLERQKLEKERHEILTQRDNAEKEWSEIRKDIDQLHIQREKLKEQREALHNEREEILQEKDTVQKEANRLRKMKYELKEGESSLRFSDQQQSQRRGQMGQDAEVLSPPQQGQSVVRDSGKELTPKTPPGPGTSTGAEVTPSRKLITSPSAGLGWLKACLFRSAEKTAGPSTAEGQAVAQGQRSQSRDVRQRLLQPSRSFNQSQIAQAVGVDAVGSVKKRVKRAKRTGPMQVVTEEARQGNSQADWTGSQMEDREAEVMSEADGGRNQEALVPEEGSKGKDAEVNDIAQTSEPRGAKGGRKRRRQPVKETSQNYLENNEESDSDTAGATRRKRRFKDIEVLSGYNGDSGLDTPTSQANTPGGGRRYNLRRSTLINTKATQAASAQSEERELSAQQERSQRKKAPPASIPEASGDLPEVSSPPPSQREVRPMTTITLSTLVIEETIVKTEVTNGIVEGNELEGNGSPEEGVGVLERASDEPVQEQHEGSQDGAVQEQQEDPQEEAVQDLQHDLQEVIGEGLLAGYEFGQGSAGVQLDARNGDGEEHDGDDGDGDEGVLEEEVVEEVEEEELEDGVEADVEEVEEGGEEVEEVEEGEDEEEVELEEDEEMEEVDEVEPEEEQSESQEEAETPKPTIGQRVWDFLIT
